# Identification of potential biomarkers and therapeutic targets for underactive bladder based on bioinformatics analysis and experimental validation

**DOI:** 10.1371/journal.pone.0335455

**Published:** 2025-11-06

**Authors:** Chen Chen, Zhuojing Hu, Yunbo Ma, Qinghua Xia, Zheng Ma, Jiangsong Li, Wei Zhao

**Affiliations:** 1 Department of Urology, Liaocheng People’s Hospital, Liaocheng, Shandong, China; 2 Medical Integration and Practice Center, Shandong University, Jinan, Shandong, China; 3 Liaocheng People’s Hospital Affiliated to Shandong First Medical University, Liaocheng, Shandong, China; 4 Department of Urology, Shandong Provincial Hospital Affiliated to Shandong First Medical University, Jinan, Shandong, China; The University of Sheffield, UNITED KINGDOM OF GREAT BRITAIN AND NORTHERN IRELAND

## Abstract

Underactive bladder (UAB) is a common disorder that significantly affects patients’ quality of life, necessitating the exploration of underlying molecular mechanisms for more effective management. This study aims to elucidate the gene expression profiles associated with UAB by employing a combination of bioinformatics analyses and experimental validation to identify pivotal hub genes and potential therapeutic targets. We accessed the GSE122060 and GSE100219 datasets from the Gene Expression Omnibus (GEO) database to identify differentially expressed genes (DEGs), followed by functional enrichment analysis, construction of a protein-protein interaction (PPI) network, screening for hub genes and assess the accuracy and diagnostic value of the hub genes with the validation dataset GSE28242. Eighty-five DEGs were identified from the GEO dataset, with functional enrichment analysis focusing primarily on biological processes like neutrophil migration, cell chemotaxis, and bacterial defense responses. Twelve key genes were identified in the PPI network using CytoHubba and MCODE plugins. Of these, C3, CLEC4E, CSF3R, CXCR2, FPR2, and IDO1 showed significant upregulation in the validation set compared to the control group. Receiver operating characteristic (ROC) curve analysis demonstrated that these six hub genes possess high diagnostic potential, with area under the curve (AUC) values greater than 0.76. Additionally, a hub gene-transcription factor (TF) interaction network, a hub gene-TF-miRNA co-regulatory network and a hub gene-drug interaction network were constructed, revealing that five TFs and five miRNAs regulate three or more hub genes. Quantitative real-time polymerase chain reaction (qRT-PCR) validation confirmed the differential expression patterns of the 12 key genes in the PPI network in TGF-β1 treated SV-HUC-1 cells. In conclusion, our findings suggest that CLEC4E, CSF3R, CXCR2, FPR2, and IDO1 can serve as promising diagnostic biomarkers for UAB, while the identified TFs and miRNAs could unveil new avenues for drug discovery and therapeutic interventions targeting UAB progression.

## Introduction

Underactive bladder (UAB), a prevalent form of lower urinary tract dysfunction, is characterized by a symptom complex indicative of detrusor underactivity (DU), typically presenting with prolonged micturition time, incomplete bladder emptying, urinary hesitancy, diminished bladder filling sensation, and reduced urine flow [1,2]. DU, a urodynamically defined condition, is marked by decreased detrusor contraction strength and/or duration, resulting in prolonged bladder emptying [3]. The prevalence of DU increases with age, affecting 9–28% of men aged 18–50 and up to 48% of men over 70 [4], while women over a certain age exhibit a prevalence ranging from 12% to 45% [5].

UAB is primarily classified into three types: myogenic, idiopathic, and neurogenic. Besides recognized causes such as nerve injury and diabetes, bladder outlet obstruction (BOO) and normal aging may also contribute to UAB [6,7]. BOO is one of the primary factors in myogenic cases, as the detrusor attempts to compensate for the increased pressure in the bladder through hyperplasia. Bladder ischemia and oxidative stress can impair cellular function and decrease bladder reactivity. These early compensatory responses may cause inflammation, eventually leading to fibrosis and irreversible changes in the extracellular matrix as the condition progresses to the decompensated phase [8,9]. Current UAB treatments are limited, often relying on invasive interventions such as abdominal pressure voiding, stoma creation, and catheterization, or pharmacological approaches that inadequately address the underlying pathophysiology. Intravesical electrical stimulation and sacral nerve modulation have demonstrated efficacy, particularly in neurogenic and idiopathic DU, as well as non-obstructive urinary retention. However, challenges remain in patient selection, complication management, and economic burden [10]. Consequently, there is an urgent need for research to identify novel biomarkers and therapeutic targets to improve UAB management and treatment outcomes.

This study aims to identify differentially expressed genes (DEGs) associated with UAB by analyzing gene expression profiles from the Gene Expression Omnibus (GEO) database. Through functional enrichment analysis and protein-protein interaction (PPI) network construction, we will elucidate key regulatory molecules, their transcription factors (TFs), miRNAs, and potential targeted drugs. Validation of hub genes using independent datasets and qRT-PCR experiments will provide novel insights and potential therapeutic targets for UAB prevention and treatment.

## Materials and methods

### Data acquisition and analysis

The gene expression profile data were downloaded from GEO database (https://www.ncbi.nlm.nih.gov/geo/), including two test sets (GSE122060, GSE100219) and one validation set (GSE28242). GEO is a public functional genomic database that includes high‐throughput gene expression data, chips, and microarrays [11]. For GSE122060, we extracted data from the bladder DU group (GSM3453947, GSM3453948, and GSM3453949) and the control group (GSM3453944, GSM3453945, and GSM3453946). For GSE100219, we extracted data from the elderly bladder dysfunctions group (GSM2674803, GSM2674804, GSM2674805, GSM2674806, GSM2674807, GSM2674808, GSM2674809, and GSM2674810) and the control group (GSM2674811, GSM2674812, GSM2674813, GSM2674814, GSM2674815, GSM2674816, GSM2674817, and GSM2674818). The GSE28242 validation set contains data from 3 experimental group samples (GSM699145, GSM699146, and GSM699147) and 5 control group samples (GSM699140, GSM699141, GSM699142, GSM699143, and GSM699144). The experimental design of the samples excluded factors such as neurogenic bladder, diabetes, and immunodeficiency. We used the GEO2R tool to analyze, standardize, and clean the data according to the sample sequencing platform. The threshold for DEGs is set at |log_2_ fold change (FC)| > 1 and *p.adj* (Benjamini & Hochberg) < 0.05. We converted the detected genes’ IDs and standardized the gene names to the official gene symbols.

### Differential expression analysis and functional clustering analysis

Use R (version 4.2.1) and related packages (https://www.r-project.org/) to analyze the data, employing the ggplot2 package for visualizing the results of differential analysis with a volcano plot, and using the ComplexHeatmap package for visualizing heatmaps. The heatmap values represent Z-score transformed gene expression levels, where the Z-score normalization was applied to the gene expression matrix. Gene clustering, performed through an unsupervised learning approach, primarily utilizes the Euclidean distance metric for hierarchical clustering analysis. For gene functional enrichment analysis, utilize the clusterProfiler package, ggplot2 package, igraph package, and ggraph package. Gene function clustering mainly involves Gene Ontology (GO) analysis and Kyoto Encyclopedia of Genes and Genomes (KEGG) pathway analysis.

### Construction of PPI networks, identification of hub genes, and module analysis

Use the STRING database (https://cn.string-db.org/) to create a PPI network for DEGs, and visualize it with Cytoscape software version 3.9.1 (https://cytoscape.org/index.html). Utilize the CytoNCA plugin to rank genes according to their interaction nodes, referred to as degree, in the network. Then, use the CytoHubba plugin to screen and extract hub genes, which identifies key nodes in the PPI network through various centrality algorithms, assessing the topological importance of nodes in the network. This is based on multiple centrality metrics including degree centrality, betweenness centrality, closeness centrality, and maximum neighborhood component, to identify the core driving factors of the regulatory network [12]. Next, use the MCODE plugin to identify highly interconnected functional submodules in the PPI network and analyze co-expressed or co-regulated gene groups.

### Validation of hub gene expression in other datasets

Conduct a group comparison analysis on the selected genes using the stats and car packages, along with information from the validation dataset. Perform diagnostic receiver operating characteristic (ROC) curve analysis with the pROC package and visualize the results using ggplot2.

### Construction of the network of interactions between TFs and hub genes

We built the TFs-hub genes interaction network using NetworkAnalyst (version 3.0, https://www.networkanalyst.ca/NetworkAnalyst/home.xhtml) platform and established interactions via the JASPAR database (http://jaspar.genereg.net). NetworkAnalyst is an online tool for comprehensive visualization and analysis of gene expression profile data [13].

### Construction of co-regulatory network of TF, miRNAs, and hub genes

Develop a regulatory network integrating TFs, miRNAs, and hub genes by utilizing TarBase v9.0 (http://microrna.gr/tarbase/) and JASPAR on NetworkAnalyst for simultaneous analysis of Gene-miRNA and TF-gene interactions. TarBase v9.0 is currently a comprehensive and authoritative database of miRNA-target interactions, focusing on integrating purely experimental validated data (CLIP-seq, degradome), providing researchers with high-confidence information on miRNA regulatory networks [14]. Construct an interaction network connecting hub genes with predicted miRNA targets via the miRWalk database (http://mirwalk.umm.uni-heidelberg.de/), applying a threshold score above 1 for miRNA predictions that may regulate hub genes. The miRWalk database integrates multiple algorithms, covers the entire genome, and offers flexible screening capabilities, making it a comprehensive platform for predicting miRNA targets [15]. Finally, visualize the results using Cytoscape.

### Construction of the drug and hub genes interaction network

The Drug Gene Interaction Database (DGIdb, https://dgidb.org/) is a comprehensive platform that combines data on drug-gene interactions, helping to identify drug targets, combinations, and repurposing opportunities [16]. Potential drugs or compounds for the hub genes were analyzed using DGIdb. The interaction network was visualized using Cytoscape.

### Cell culture, model establishment, RNA extraction, reverse transcription, and qRT-PCR

**Fig 1:**
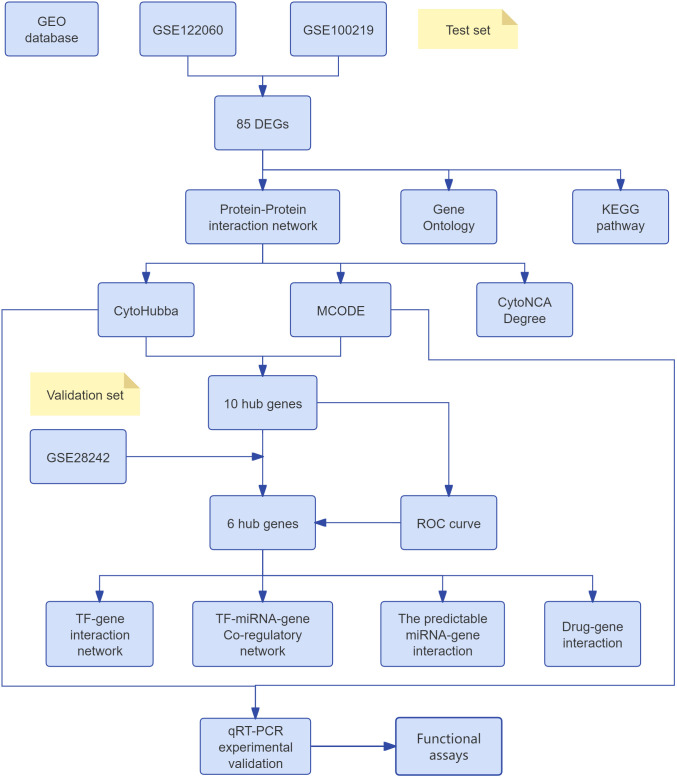
Flow chart.

The human urinary tract epithelial immortalized cell line SV-HUC-1 was purchased from Shanghai Zhong Qiao Xin Zhou Biotechnology Co., Ltd. The transforming growth factor-beta 1 (TGF-β1) cytokine was obtained from Wuhan Pricella Biotechnology Co., Ltd. After seeding the cells into six-well plates and allowing them to adhere, TGF-β1 was added at a dose of 10 ng/mL for induction. The cells were then treated for 48–72 hours to establish an in vitro model of bladder dysfunction. Cells were cultured with penicillin/streptomycin at 37°C in 5% CO_2_. RNA was extracted using an Accurate Biotechnology kit, and reverse transcription was done with Evo M-MLV RT Premix. qPCR was performed using SYBR® Green Premix Pro Taq HS kit and amplified in LightCycler 480II, normalizing RNA expression with β-actin. Gene expression was calculated using the 2^−ΔΔCt^ method, with primer sequences in Table 1. The overall flowchart of the research is shown in Fig 1.

**Table 1:** The primer sequences used are listed.

*Gene Symbol*	*Primer Sequence(5’-3’)*
C3	F: CCTCAGGATGCCAAGAACACT
	R: GGTCATCTGTGTCTGGAGCAA
CCL20	F:CAACTTTGACTGCTGTCTTGGATA
	R: GCCGTGTGAAGCCCACAATA
CLEC4E	F: GTAGGGGAGCCCAACAACATA
	R: TTGCCTTGGGTTTGAAGAGTC
CSF3R	F: TCTCAAGAAATGGCTCCCTCC
	R: CTGAGCGTTGGTCCAGAAGA
CXCL13	F: CCAAGGTGTTCTGGAGGTCTAT
	R: TGGACAACCATTCCCACGG
CXCR2	F: TCAACCCCCTCATCTACGCC
	R: GTGCCCTGAAGAAGAGCCAAC
FPR2	F: TTGCCGATGTCCATTGTTGC
	R: AGAAAGAAGCCACCACAGCA
IDO1	F: AGTCAAATCCCTCAGTCCGTG
	R: TTTCACACAGGCGTCATAAGC
LCN2	F: GTTCACGCTGGGCAACATTA
	R: GATTGGGACAGGGAAGACGA
PLA2G2A	F: AAGACCCTCCTACTGTTGGCA
	R: ATAACTGAGTGCGGCTTCCTT
S100A9	F: TGGAGGACCTGGACACAAATG
	R: ACCCTCGTGCATCTTCTCGT
SERPINB2	F: TCAGAACCCCAGGCAGTAGA
	R: GACAGCATTCACCAGGACCA
GAPDH	F: GCACCGTCAAGGCTGAGAAC
	R: TGGTGAAGACGCCAGTGGA
β-actin	F: CATGTACGTTGCTATCCAGGC
	R: CTCCTTAATGTCACGCACGAT

### Lentiviral transfection

For the generation of lentiviral particles targeting FPR2 knockdown, HEK293T cells were co-transfected with three essential plasmids: the FPR2-targeting short hairpin RNA (shRNA) vector plasmid and two viral packaging plasmids (Helper 1.0 and Helper 2.0). Viral supernatants were collected 48 hours post-transfection, followed by concentration, purification, and subsequent quality assessment. Three specific RNA interference sequences were designed: PSC94228−1 (GACTACAGTAACTATTCCAAA), PSC94229−1 (GCCACATTACCATTCCTCATT), and PSC94230−1 (GGCCAAGACTTCCGAGAGAGA). The lentiviral vector (LV) GV493 was utilized, featuring the following element configuration: hU6-MCS-CBh-gcGFP-IRES-puromycin, with AgeI and EcoRI serving as cloning sites. The negative control virus (CON313) contained the scrambled sequence TTCTCCGAACGTGTCACGT. All lentiviral constructs were procured from Shanghai Genechem Co., Ltd. (China). A schematic representation of the LV architecture is provided in S1 Fig.

### Functional cell assays

The CCK-8 assay was employed to assess cell proliferation. Lentivirus-stably transfected cells were seeded at a density of 4 × 10^4 cells/mL in a 96-well plate, with 100 μL of cell suspension added to each well. Five replicates were prepared for each experimental group, followed by incubation at 37°C in a 5% CO₂ atmosphere. Under light-protected conditions, the CCK-8 solution was prepared by mixing incomplete culture medium with CCK-8 reagent (Dojindo Molecular Technologies, Inc., Japan) at a 10:1 ratio. The plate was further incubated for 2 hours, and absorbance at 450 nm was measured using a microplate reader at 24, 48, and 72 hours to generate a standard curve. For the transwell migration assay, a 24-well plate was utilized, with 600 μL of culture medium containing 20% serum added to the lower chamber of the transwell insert (Corning Incorporated, NY, USA). Subsequently, 200 μL of cell suspension was added to the upper chamber, and the plate was incubated under standard conditions. After 48 hours, the insert was removed, fixed with 4% paraformaldehyde, washed, and stained with crystal violet. The filter membrane was then detached, mounted on a glass slide using neutral gum, air-dried, and imaged under a microscope for analysis.

### Statistical analysis

All statistical evaluations were conducted utilizing R software (version 4.2.1) in conjunction with GraphPad Prism 9 software. The determination of statistical significance for differences between groups and among multiple groups was carried out through the application of the t-test and one-way analysis of variance (ANOVA), respectively. *P* < 0.05 was considered to indicate statistical significance. The following *p*-values were considered: *: *p* < 0.05, **: *p* < 0.01, and ***: *p* < 0.001.

## Results

### Identification of DEGs in UAB

**Fig 2:**
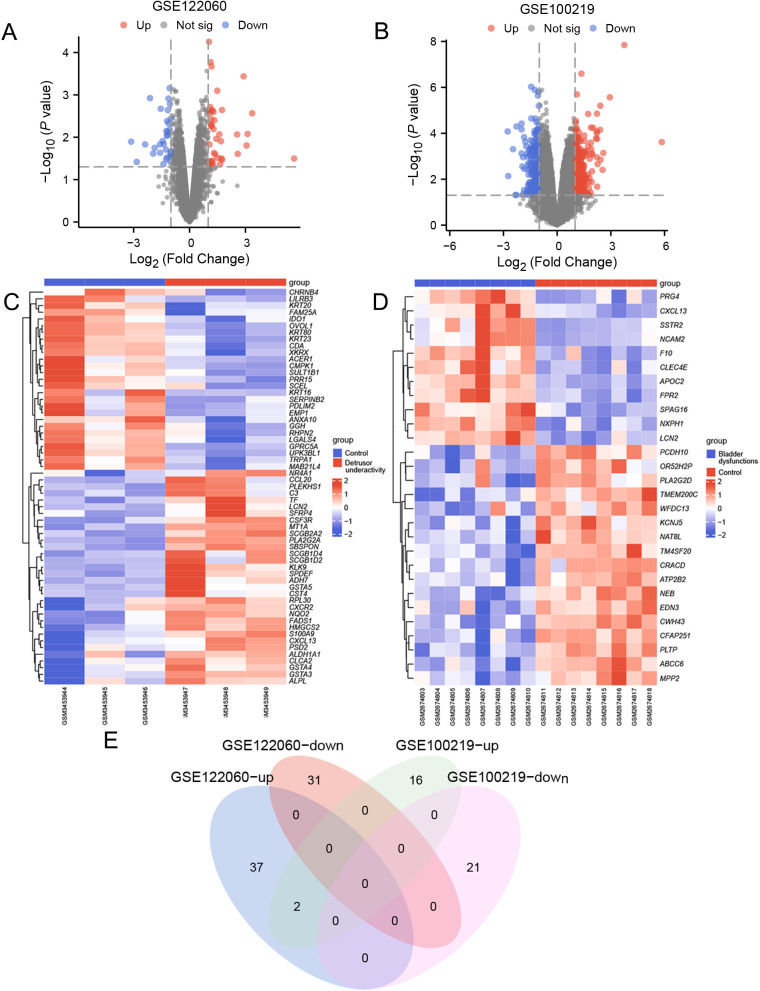
Identification of differentially expressed genes (DEGs). **(A-B)** Volcano plot of DEGs between datasets GSE122060 and GSE100219. Red dots: upregulated, blue dots: downregulated. Parameter settings are |log_2_FC| > 1 and *p*-value (FDR correction) < 0.05. **(C-D)** Heatmap of DEGs in datasets GSE122060 and GSE100219. Rows represent genes; columns represent samples. Expression values were row-normalized (Z-score) based on log_2_(TPM + 1) data. Hierarchical clustering (Euclidean distance, average linkage) was applied to genes. **(E)** Venn diagram of upregulated and downregulated genes in two datasets.

The GSE122060 dataset has 39 up-regulated genes and 31 down-regulated genes (|log_2_FC| > 1 and *p.adj* < 0.05) (Fig 2A), while the GSE100219 dataset has 18 up-regulated genes and 21 down-regulated genes (|log_2_FC| > 1 and *p.adj* < 0.05) (Fig 2B). The corresponding heatmaps are shown in Fig 2C and 2D. The Venn diagram shows the upregulated and downregulated DEGs in two datasets (Fig 2E), with two common upregulated genes: CXCL13 and LCN2. CXCL13, a B cell chemokine, and its receptor CXCR5 form a chemotactic axis whose aberrant activation drives B cell-mediated immune responses, thereby promoting inflammatory processes and endothelial injury, which are implicated in the pathophysiology of interstitial cystitis/bladder pain syndrome. Pharmacological inhibition of the CXCL13/CXCR5 axis using the selective inhibitor TAK-799 has been shown to attenuate bladder inflammation and restore bladder function in murine models of autoimmune cystitis [17]. LCN2, a neutrophil-associated protein, exerts antimicrobial effects through its ability to sequester bacterial siderophores, thereby inducing iron deprivation and representing a novel mechanism of host defense against infection [18]. In the urogenital tract, LCN2 is secreted by urethral mucosa and has been demonstrated to mitigate Escherichia coli infection in bladder epithelial cells by modulating JAK/STAT pathway activation, particularly under hyperglycemic conditions [19,20]. The concurrent upregulation of both CXCL13 and LCN2 suggests potential synergistic interactions between B cells and neutrophils in mediating inflammatory and immune responses during the development of UAB.

### Functional enrichment analysis of DEGs

**Fig 3:**
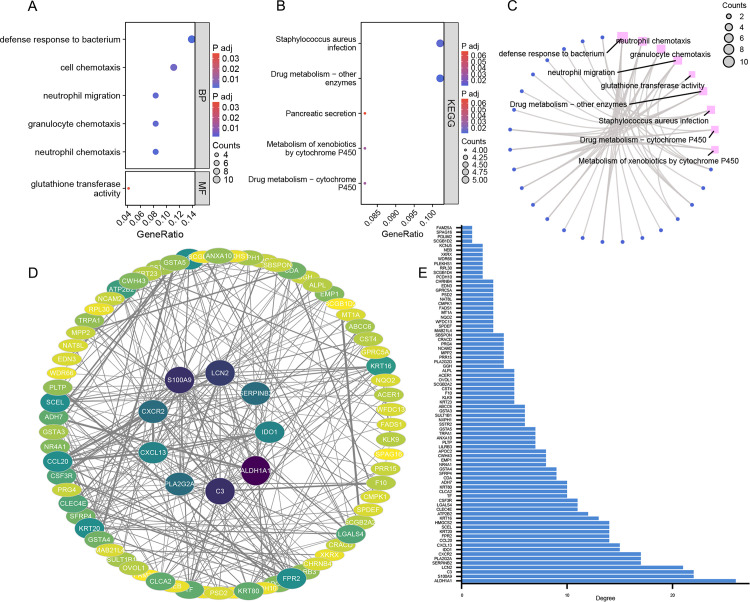
Functional enrichment analysis of DEGs, protein-protein interaction (PPI) network construction. **(A)** Gene Ontology (GO) analysis of DEGs, including the major biological processes (BP) and molecular functions (MF) involved. **(B)** Kyoto Encyclopedia of Genes and Genomes (KEGG) pathway analysis of DEGs. **(C)** The circular network diagram of functional enrichment analysis. **(D)** The PPI network constructed from DEGs, with 9 genes in the inner circle having a degree ≥ 15. The color intensity of the genes corresponds to their degree values. **(E)** Bar chart of the interaction number of each DEG.

We combined the DEGs from the two datasets to conduct a thorough analysis. After organizing the data and converting it to official gene symbols, we identified a total of 85 DEGs. The specific gene list can be found in Supporting information S1 Table. GO enrichment analysis of DEGs identified the top five significantly enriched biological processes, including neutrophil chemotaxis, granulocyte chemotaxis, neutrophil migration, cell chemotaxis, and the defense response to bacterium (*p.adj *< 0.01). The sustained chemotaxis of neutrophils toward bladder tissue induces chronic inflammation, which may contribute to bladder wall tissue damage and fibrotic remodeling, ultimately leading to UAB and impaired detrusor muscle contractility. Furthermore, neutrophil-derived inflammatory mediators disrupt bladder neural signaling, resulting in neuro-immune regulatory dysfunction, which may manifest as diminished bladder sensation and attenuated contraction signals. Molecular function analysis revealed significant enrichment in glutathione transferase activity (*p.adj *< 0.05) (Fig 3A and 3C). Reduced glutathione S-transferase activity compromises the clearance of reactive oxygen species (ROS) in bladder tissue, leading to oxidative stress accumulation, mitochondrial dysfunction in detrusor cells, and impaired neurotransmission, collectively contributing to diminished bladder contractile function. KEGG pathway analysis demonstrated that DEGs were predominantly associated with Staphylococcus aureus infection, Drug metabolism-other enzymes, Metabolism of xenobiotics by cytochrome P450, and Drug metabolism – cytochrome P450 (*p.adj *< 0.05) (Fig 3B and 3C). Recurrent bacterial infections and persistent colonization promote immune tolerance, resulting in neutrophil exhaustion/deactivation, immunosuppressive cell infiltration, bacterial immune evasion, and the establishment of a chronic inflammatory cycle, with prolonged inflammation ultimately culminating in UAB. The detailed information can be found in S2 Table.

### Construction of PPI network and identification of hub genes

**Fig 4:**
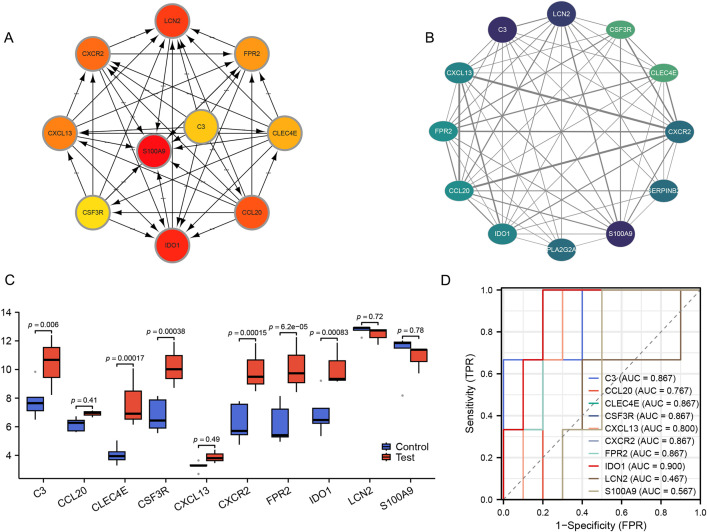
Hub gene identification, and GEO dataset validation. **(A)** The top 10 hub genes identified in the PPI network through the Cytohubba plugin, with the color intensity of the genes corresponding to their score values. **(B)** The functional clustering module extracted from the PPI network through MCODE contain 12 genes, with the color intensity of the genes corresponding to their degree values. **(C)** The mRNA expression of 10 hub genes was determined from the GSE28242 validation dataset, six genes have significant expression differences. **(D)** Diagnostic value of 10 hub genes analyzed with ROC curves. AUC, area under the ROC curve. ns, no significant difference.

The PPI network of DEGs comprises 84 nodes and 296 edges (Fig. 3D), with nine genes exhibiting an interaction degree of 15 or higher: ALDH1A1, S100A9, C3, LCN2, SERPINB2, PLA2G2A, CXCR2, IDO1, and CXCL13 (Fig 3D). Genes with a degree value ≥ 15, representing the top 10% of the PPI network, were selected to filter low-connectivity nodes, consistent with the scale-free network property characterized by a hub distribution of highly connected nodes, thereby retaining biologically significant hub genes. The interaction counts for each gene are presented in Fig 3E and S3 Table. Using CytoHubba, the top 10 regulatory hub genes were identified: C3, CCL20, CLEC4E, CSF3R, CXCL13, CXCR2, FPR2, IDO1, LCN2, and S100A9 (Fig 4A). Furthermore, MCODE analysis revealed a subnetwork of 12 DEGs: C3, CCL20, CLEC4E, CSF3R, CXCL13, CXCR2, FPR2, IDO1, LCN2, PLA2G2A, S100A9, and SERPINB2 (Fig 4B). CytoHubba evaluates gene importance based on global connectivity, emphasizing the hub nature of genes within the entire network, while MCODE identifies highly interconnected subnetworks (functional modules) through a density clustering algorithm, uncovering gene groups with functional synergy. By integrating both algorithms, hub genes exhibiting characteristics of core regulatory factors and functional modules were identified, providing a comprehensive understanding of the network’s biological features at multiple levels. Through a comparative analysis of hub genes identified via CytoHubba and overlapping genes derived from the MCODE module, ten DEGs were identified as key hub genes for further investigation. These genes, ranked according to the Maximal Clique Centrality (MCC) algorithm within CytoHubba, include S100A9, IDO1, LCN2, CCL20, CXCR2, CXCL13, FPR2, CLEC4E, C3, and CSF3R.

### Verification and diagnostic value of the 10 hub genes expression

Expression analysis of 10 hub genes was conducted using the validation dataset GSE28242, revealing significant upregulation (*p* < 0.05) in six genes compared to the control group. These genes were ranked based on their p-values in ascending order of significance: FPR2 (*p* = 6.2e − 05, |log2FC| = 1.73), CXCR2 (*p* = 0.00015, |log2FC| = 1.14), CLEC4E (*p* = 0.00017, |log2FC| = 2.4), CSF3R (*p* = 0.00038, |log2FC| = 1.11), IDO1 (*p* = 0.00083, |log2FC| = 1.95), and C3 (*p* = 0.006, |log2FC| = 2.55) (Fig 4C). To evaluate the diagnostic potential of these genes, ROC curve analysis was performed, with the AUC serving as the metric. The analysis demonstrated that, with the exception of S100A9 and LCN2, all genes exhibited AUC values exceeding 0.76: IDO1 [AUC: 0.900 (0.72–1.00)], CLEC4E [AUC: 0.867 (0.65–1.00)], CSF3R [AUC: 0.867 (0.65–1.00)], CXCR2 [AUC: 0.867 (0.65–1.00)], FPR2 [AUC: 0.867 (0.65–1.00)], C3 [AUC: 0.867 (0.584–1.00)], CXCL13 [AUC: 0.800 (0.551–1.00)], and CCL20 [AUC: 0.767 (0.50–1.00)] (Fig 4D). Based on the combined results of differential expression and ROC curve analyses, six hub genes—C3, CLEC4E, CSF3R, CXCR2, FPR2, and IDO1—were identified as potential biomarkers for UAB.

### TF-gene interaction

**Fig 5:**
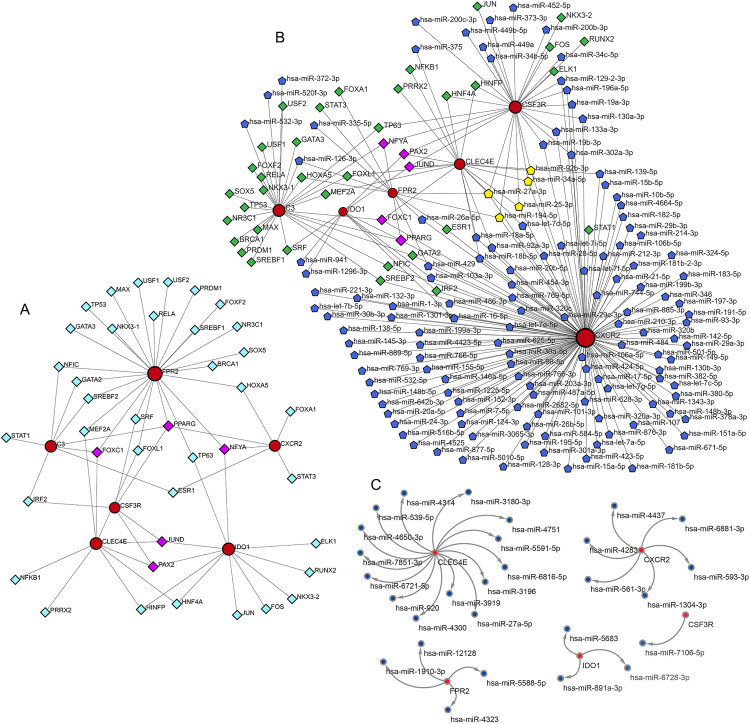
The interaction network between transcription factors (TFs) and six hub genes, the co-regulatory network of TF-miRNA-hub genes, and miRNA target prediction. **(A)** The TF-hub genes interaction network. The red circles represent hub genes, the light blue diamonds represent TFs interacting with hub genes, and the purple diamonds represent TFs interacting with no less than three hub genes. **(B)** The interaction network between TF-miRNA and six hub genes. The red circle represents the hub gene, the blue pentagon represents the miRNAs that interact with the hub gene, the yellow pentagon represents the miRNAs that interact with no less than three hub genes, the green diamond represents the TFs that interact with the hub gene, and the purple diamond represents the TFs that interact with no less than three hub genes. **(C)** The predictable miRNAs that interact with hub genes.

Six candidate biomarkers were analyzed for TF-gene interactions using the JASPAR database on NetworkAnalyst, revealing a network of 46 nodes and 63 edges (Fig 5A). PPARG interacts with FPR2, C3, CSF3R, and CXCR2; NFYA with IDO1, FPR2, and CXCR2; FOXC1 with CLEC4E, FPR2, and C3; and JUND and PAX2 with CSF3R, CLEC4E, and IDO1. More details on the TFs are in S4 Table.

### TF-miRNA coregulatory network and Hub gene-miRNA interactions network

The network co-regulated by TFs, miRNAs and hub genes includes six hub genes, 136 miRNAs, and 40 TFs, totaling 182 nodes and 227 edges (Fig 5B, S5 Table). The degrees of the six hub genes—CXCR2, CSF3R, C3, CLEC4E, FPR2, and IDO1—are 128, 31, 28, 19, 12, and 9, respectively. hsa-miR-27a-3p regulates four hub genes: CSF3R, CLEC4E, FPR2, and CXCR2. hsa-miR-194-5p regulates three hub genes, namely CSF3R, FPR2, and CXCR2. hsa-miR-25-3p, hsa-miR-34a-5p, and hsa-miR-92b-3p each regulate three hub genes, which are CSF3R, CLEC4E, and CXCR2 (Fig 5B). To identify miRNAs that can predict the six hub genes, we switched to the miRWalk database for miRNA target prediction. The predicted miRNAs that regulate the five remaining hub genes, excluding C3, are shown in Fig 5C.

### Drug-gene interaction network

The drug-gene interaction network includes five hub genes and their drug interactions, with FPR2 and CHEMBL1290139 having the highest score of 8.70. LINRODOSTAT scores 7.46 with IDO1, while NAVARIXIN scores 4.35 with CXCR2. TBO-FILGRASTIM, PEGFILGRASTIM, and MAXY-G34 score 3.87 with CSF3R. COMPSTATIN has the strongest interaction with C3 at 11.60 (S2 Fig, S6 Table).

### qRT-PCR experimental validation

**Fig 6:**
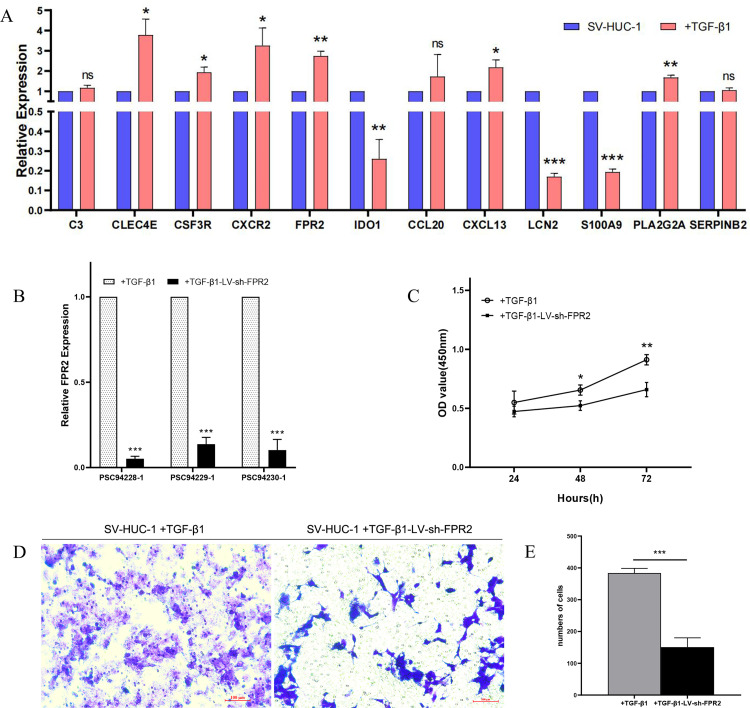
qPCR validation of DEGs and functional assays. **(A)** qPCR validation of DEGs identified through CytoHubba and MCODE analysis in the PPI network. Among the six hub genes, except for C3 which showed no statistical difference, CLEC4E, CSF3R, CXCR2, and FPR2 were upregulated in the disease group compared to the control group, while IDO1 was downregulated. **(B)** Assessment of FPR2 knockdown efficiency by qPCR. **(C)** CCK-8 assay for assessing the viability of SV-HUC-1 cells. **(D, E)** Transwell assay to determine the migration ability of SV-HUC-1 cells (Scale bar, 100 μm). *: *p* < 0.05, **: *p* < 0.01, ***: *p* < 0.001. ns: no significance.

We conducted qRT-PCR validation on all genes identified by the CytoHubba and MCODE analyses within the PPI network. We observed that, except for C3, CCL20, and SERPINB2, the other nine genes exhibited significant statistical differences when comparing TGF-β1-treated SV-HUC-1 cells to the control group. Among these, CLEC4E, CSF3R, CXCR2, FPR2, CXCL13, and PLA2G2A showed upregulation in the disease group, whereas IDO1, LCN2, and S100A9 showed downregulation (Fig 6A). In conclusion, the qRT-PCR experiments confirmed our earlier findings. CLEC4E, CSF3R, CXCR2, FPR2, and IDO1 may serve as potential diagnostic biomarkers for UAB.

### Knockdown of FPR2 expression inhibits the proliferation and migration rates of TGF-β1-treated SV-HUC-1 cells

To further elucidate the functional role of hub genes, we integrated data from the validation set with qPCR results to perform lentivirus-mediated knockdown of FPR2, the most significantly expressed gene in the test group, and subsequently assessed changes in functional phenotypes. Initially, the transfection efficiency of three FPR2-specific shRNA sequences was evaluated via qPCR, revealing that PSC94228–1 exhibited the highest knockdown efficiency, exceeding 90% (Fig 6B). This sequence was therefore selected for subsequent functional assays. Functional assays demonstrated that FPR2 suppression (LV-sh-FPR2) significantly attenuated the proliferative capacity of TGF-β1-stimulated SV-HUC-1 cells, as evidenced by CCK-8 analysis (Fig 6C). Furthermore, Transwell migration assays revealed that FPR2 silencing markedly inhibited the migratory activity of TGF-β1-induced SV-HUC-1 cells (Fig 6D, 6E). As a G protein-coupled receptor primarily involved in inflammatory and immunomodulatory processes, FPR2 deficiency has been shown to mitigate inflammation and oxidative stress [21–24]. Mechanistically, FPR2 has been reported to directly interact with TGF-β-activated kinase 1 (TAK1), a pivotal kinase in the TGF-β signaling cascade, suggesting its potential involvement in downstream TGF-β receptor signaling [25]. Our findings demonstrate that FPR2 inhibition attenuates TGF-β-induced aberrant epithelial proliferation in bladder cells, reduces disordered epithelial cell migration, preserves epithelial barrier integrity, and partially mitigates the risk of TGF-β-mediated bladder fibrosis.

## Discussion

UAB presents with a multifactorial etiology, and its nonspecific clinical manifestations often overlap with those of overactive bladder and BOO, leading to frequent misdiagnosis [26,27]. The molecular mechanisms underlying UAB remain poorly understood, and the current therapeutic landscape is limited by the absence of disease-specific diagnostic biomarkers, underscoring the urgent need for the development of more precise diagnostic and therapeutic strategies [28,29]. In this study, we employed bioinformatics approaches to investigate the gene expression profile associated with UAB, aiming to identify and validate potential hub genes and therapeutic targets through the integration of multiple public databases. Notably, the availability of microarray and high-throughput sequencing data specific to UAB is extremely limited. Our analysis focused on two datasets: GSE122060, which examines DU, and GSE100219, which addresses age-related bladder dysfunction. The UAB-related factors identified in these datasets primarily emphasize non-neurogenic aspects rather than DU resulting from denervation [30,31].

GO enrichment analysis of the DEGs revealed significant involvement in neutrophil chemotaxis, migration, and defense responses, while KEGG pathway analysis highlighted associations with infection and drug metabolism. These findings suggest a complex interplay between inflammatory responses and UAB pathogenesis. Bladder dysfunction can arise even in the absence of overt pathogen infection, primarily driven by sterile inflammation, which may compromise the integrity of the urinary epithelial barrier and facilitate bacterial invasion. Evidence indicates that the sterile inflammatory response mediated by the NLRP3 inflammasome contributes to UAB development [32]. Patients with DU exhibit pronounced dysfunction of the urinary tract epithelium, increased suburothelial inflammation, and apoptosis in the bladder mucosa [33]. The inflammatory cascade in UAB may be driven by several key mechanisms, including histamine release from bladder wall mast cells, macrophage polarization leading to the production of IL-6 and TNF-α, inhibition of calcium channel function in detrusor cells, and mitochondrial dysfunction. The findings from the comprehensive enrichment analysis reveal two critical mechanistic pathways underlying the pathogenesis of UAB: (1) recurrent inflammation induced by bacterial immune evasion through neutrophil chemotaxis, and (2) impaired glutathione transferase activity leading to oxidative stress accumulation, which subsequently causes detrusor and neuronal injury. These pathways collectively contribute to the development of UAB.

Validation of hub gene expression using the GSE28242 dataset revealed a significant upregulation of several genes, suggesting their potential utility as reliable biomarkers for UAB. The AUC values exceeding 0.76 for most hub genes underscore their robust diagnostic potential. To further corroborate these findings, we assessed the expression levels of these hub genes in an in vitro bladder cell model treated with TGF-β1. The histological manifestations of UAB in the bladder are influenced by multiple factors, including increased bladder weight, wall thickening, inflammation, and fibrosis [34]. Studies have demonstrated that urinary TGF-β1 levels rise significantly following the transition of the obstructive bladder to the decompensated phase, with a positive correlation observed between TGF-β1 mRNA levels in detrusor tissue and urinary TGF-β1 concentrations [35]. Elevated TGF-β1 expression has been consistently observed in UAB and BOO murine models [36,37]. The TGF-β1/Smad3 signaling pathway has been implicated in the regulation of connexin 43 (CX43) expression, contributing to bladder dysfunction in rats [38]. Furthermore, TGF-β1-induced SV-HUC-1 cells have been widely utilized as an in vitro model for studying bladder dysfunction [39–41]. In this study, we validated the expression of relevant hub genes by establishing a TGF-β1-induced SV-HUC-1 bladder fibrosis model. Experimental results demonstrated that 9 out of 12 key genes identified from the PPI network exhibited significant differential expression, thereby validating our bioinformatics analysis. Through comprehensive integration of expression differentials from the validation dataset with ROC curve analysis, we identified CLEC4E, CSF3R, CXCR2, FPR2, and IDO1 as possessing diagnostic potential in UAB. Among the six hub genes analyzed in the validation cohort, all except IDO1 exhibited consistent expression profiles when compared with both the training cohort and experimental validation results. Notably, IDO1 demonstrated downregulation in both the training cohort and qPCR experimental validation, contrasting with its upregulation observed in the validation cohort. This observed discrepancy may be mechanistically explained by the potential inhibitory effect of TGF-β1 on IDO1 transcriptional activation, mediated through the suppression of signal transducer and activator of transcription 1 (STAT1) phosphorylation and subsequent inhibition of interferon-gamma (IFN-γ) signaling. Furthermore, the chronic progression of UAB, characterized by diminished local immune cell infiltration, may contribute to the attenuation of IFN-γ signaling, potentially leading to the downregulation of IDO1 transcription [42–44]. The associated TFs and miRNAs were identified as potential regulatory nodes and therapeutic targets.

This study is subject to several limitations. Firstly, the sample size of both the training and validation cohorts is constrained, compounded by inherent heterogeneity and batch effects. Future investigations should incorporate larger patient cohorts to enhance statistical power and enable robust repeated validation. Secondly, the analytical methodologies exhibit algorithm dependency, and the network and enrichment analyses are confined to existing knowledge bases, potentially overlooking novel pathways or suffering from incomplete annotations. Furthermore, the TF-miRNA interactions are computationally inferred and require experimental validation. Thirdly, the experimental design is restricted to an in vitro bladder model, necessitating complementary in vivo animal studies to elucidate downstream pathways associated with hub genes and key targets. Subsequent research should prioritize expanded patient cohorts and prospective validation studies to facilitate potential clinical translation.

## Conclusion

In conclusion, this investigation systematically identified critical genes implicated in UAB pathogenesis through comprehensive mining of publicly available genomic databases, followed by the exploration of associated TFs, miRNAs, and their respective therapeutic targets. Subsequent validation using independent datasets and experimental approaches substantiated the biological significance of these molecular markers. The identified genes may serve as potential biomarkers for early diagnostic applications and the development of personalized therapeutic interventions. Future studies should focus on integrating these molecular insights with clinical parameters to enhance the mechanistic understanding and clinical management of bladder dysfunction.

## Supporting information

S1 Table List of 85 DEGs. (DOCX)

S2 Table Results of GO analysis and KEGG pathway analysis of DEGs (*p* < 0.05). (DOCX)

S3 Table The number of interactions for each gene in the PPI network. (DOCX)

S4 Table TFs that interacts with 6 seed proteins. (DOCX)

S5 Table Specific information list of the interaction network between TF, miRNA, and hub genes. (DOCX)

S6 Table List of specific information about the drugs that interact with hub genes. (DOCX)

S1 Fig Schematic representation of the structural components comprising the lentiviral vector system employed for FPR2 gene knockdown. (JPG)

S2 Fig The interaction network between drugs and hub genes. Red nodes represent hub genes, gray nodes represent corresponding drugs or molecular compounds.
(JPG)
