# Supplementary material for: Identification of potential biomarkers and therapeutic targets for underactive bladder based on bioinformatics analysis and experimental validation
Source: PLoS One. 2025 Nov 6;20(11):e0335455. doi: 10.1371/journal.pone.0335455 (PMC12591491; doi:10.1371/journal.pone.0335455)
Supplement: S1 Table — (DOCX) [file pone.0335455.s001.docx]

| ***ID*** | ***Gene*** | ***Description*** |
| --- | --- | --- |
| 368 | ABCC6 | ATP binding cassette subfamily C member 6 |
| 125981 | ACER1 | alkaline ceramidase 1 |
| 131 | ADH7 | alcohol dehydrogenase 7 (class IV), mu or sigma polypeptide |
| 216 | ALDH1A1 | aldehyde dehydrogenase 1 family member A1 |
| 249 | ALPL | alkaline phosphatase, biomineralization associated |
| 11199 | ANXA10 | annexin A10 |
| 344 | APOC2 | apolipoprotein C2 |
| 491 | ATP2B2 | ATPase plasma membrane Ca2+ transporting 2 |
| 718 | C3 | complement C3 |
| 6364 | CCL20 | C-C motif chemokine ligand 20 |
| 978 | CDA | cytidine deaminase |
| 144406 | CFAP251 | cilia and flagella associated protein 251 |
| 1143 | CHRNB4 | cholinergic receptor nicotinic beta 4 subunit |
| 9635 | CLCA2 | chloride channel accessory 2 |
| 26253 | CLEC4E | C-type lectin domain family 4 member E |
| 51727 | CMPK1 | cytidine/uridine monophosphate kinase 1 |
| 57482 | CRACD | capping protein inhibiting regulator of actin dynamics |
| 1441 | CSF3R | colony stimulating factor 3 receptor |
| 1472 | CST4 | cystatin S |
| 80157 | CWH43 | cell wall biogenesis 43 C-terminal homolog |
| 10563 | CXCL13 | C-X-C motif chemokine ligand 13 |
| 3579 | CXCR2 | C-X-C motif chemokine receptor 2 |
| 1908 | EDN3 | endothelin 3 |
| 2012 | EMP1 | epithelial membrane protein 1 |
| 2159 | F10 | coagulation factor X |
| 3992 | FADS1 | fatty acid desaturase 1 |
| 643161 | FAM25A | family with sequence similarity 25 member A |
| 2358 | FPR2 | formyl peptide receptor 2 |
| 8836 | GGH | gamma-glutamyl hydrolase |
| 9052 | GPRC5A | G protein-coupled receptor class C group 5 member A |
| 2940 | GSTA3 | glutathione S-transferase alpha 3 |
| 2941 | GSTA4 | glutathione S-transferase alpha 4 |
| 221357 | GSTA5 | glutathione S-transferase alpha 5 |
| 3158 | HMGCS2 | 3-hydroxy-3-methylglutaryl-CoA synthase 2 |
| 3620 | IDO1 | indoleamine 2,3-dioxygenase 1 |
| 3762 | KCNJ5 | potassium inwardly rectifying channel subfamily J member 5 |
| 284366 | KLK9 | kallikrein related peptidase 9 |
| 3868 | KRT16 | keratin 16 |
| 54474 | KRT20 | keratin 20 |
| 25984 | KRT23 | keratin 23 |
| 144501 | KRT80 | keratin 80 |
| 3934 | LCN2 | lipocalin 2 |
| 3960 | LGALS4 | galectin 4 |
| 11025 | LILRB3 | leukocyte immunoglobulin like receptor B3 |
| 79919 | MAB21L4 | mab-21 like 4 |
| 4355 | MPP2 | MAGUK p55 scaffold protein 2 |
| 4489 | MT1A | metallothionein 1A |
| 339983 | NAT8L | N-acetyltransferase 8 like |
| 4685 | NCAM2 | neural cell adhesion molecule 2 |
| 4703 | NEB | nebulin |
| 4835 | NQO2 | N-ribosyldihydronicotinamide:quinone dehydrogenase 2 |
| 3164 | NR4A1 | nuclear receptor subfamily 4 group A member 1 |
| 30010 | NXPH1 | neurexophilin 1 |
| 81261 | OR52H2P | olfactory receptor family 52 subfamily H member 2 |
| 5017 | OVOL1 | ovo like transcriptional repressor 1 |
| 57575 | PCDH10 | protocadherin 10 |
| 64236 | PDLIM2 | PDZ and LIM domain 2 |
| 5320 | PLA2G2A | phospholipase A2 group IIA |
| 26279 | PLA2G2D | phospholipase A2 group IID |
| 79949 | PLEKHS1 | pleckstrin homology domain containing S1 |
| 5360 | PLTP | phospholipid transfer protein |
| 10216 | PRG4 | proteoglycan 4 |
| 222171 | PRR15 | proline rich 15 |
| 84249 | PSD2 | pleckstrin and Sec7 domain containing 2 |
| 85415 | RHPN2 | rhophilin Rho GTPase binding protein 2 |
| 6156 | RPL30 | ribosomal protein L30 |
| 6280 | S100A9 | S100 calcium binding protein A9 |
| 157869 | SBSPON | somatomedin B and thrombospondin type 1 domain containing |
| 8796 | SCEL | sciellin |
| 10647 | SCGB1D2 | secretoglobin family 1D member 2 |
| 404552 | SCGB1D4 | secretoglobin family 1D member 4 |
| 4250 | SCGB2A2 | secretoglobin family 2A member 2 |
| 5055 | SERPINB2 | serpin family B member 2 |
| 6424 | SFRP4 | secreted frizzled related protein 4 |
| 79582 | SPAG16 | sperm associated antigen 16 |
| 25803 | SPDEF | SAM pointed domain containing ETS transcription factor |
| 6752 | SSTR2 | somatostatin receptor 2 |
| 27284 | SULT1B1 | sulfotransferase family 1B member 1 |
| 7018 | TF | transferrin |
| 79853 | TM4SF20 | transmembrane 4 L six family member 20 |
| 645369 | TMEM200C | transmembrane protein 200C |
| 8989 | TRPA1 | transient receptor potential cation channel subfamily A member 1 |
| 100134938 | UPK3BL1 | uroplakin 3B like 1 |
| 164237 | WFDC13 | WAP four-disulfide core domain 13 |
| 402415 | XKRX | XK related X-linked |
