# Supplementary material for: Identification of potential biomarkers and therapeutic targets for underactive bladder based on bioinformatics analysis and experimental validation
Source: PLoS One. 2025 Nov 6;20(11):e0335455. doi: 10.1371/journal.pone.0335455 (PMC12591491; doi:10.1371/journal.pone.0335455)
Supplement: S2 Table — (DOCX) [file pone.0335455.s002.docx]

| ***ONTOLOGY*** | ***ID*** | ***Description*** | ***BgRatio*** | ***pvalue*** | ***p.adjust*** | ***qvalue*** | ***geneID*** | ***Count*** |
| --- | --- | --- | --- | --- | --- | --- | --- | --- |
| BP | GO:0042742 | defense response to bacterium | 364/18800 | 1.20776E-06 | 0.001579751 | 0.001343792 | CCL20/CLEC4E/CXCL13/FPR2/LCN2/PLA2G2A/RPL30/S100A9/TF/WFDC13 | 10 |
| BP | GO:0030593 | neutrophil chemotaxis | 106/18800 | 3.21861E-06 | 0.002104974 | 0.001790566 | CCL20/CSF3R/CXCL13/CXCR2/EDN3/S100A9 | 6 |
| BP | GO:0071621 | granulocyte chemotaxis | 128/18800 | 9.57768E-06 | 0.0031319 | 0.002664106 | CCL20/CSF3R/CXCL13/CXCR2/EDN3/S100A9 | 6 |
| BP | GO:1990266 | neutrophil migration | 128/18800 | 9.57768E-06 | 0.0031319 | 0.002664106 | CCL20/CSF3R/CXCL13/CXCR2/EDN3/S100A9 | 6 |
| BP | GO:0060326 | cell chemotaxis | 315/18800 | 2.68115E-05 | 0.005121675 | 0.00435668 | CCL20/CSF3R/CXCL13/CXCR2/EDN3/FPR2/NR4A1/S100A9 | 8 |
| BP | GO:0097529 | myeloid leukocyte migration | 229/18800 | 2.72985E-05 | 0.005121675 | 0.00435668 | CCL20/CSF3R/CXCL13/CXCR2/EDN3/FPR2/S100A9 | 7 |
| BP | GO:0097530 | granulocyte migration | 154/18800 | 2.74157E-05 | 0.005121675 | 0.00435668 | CCL20/CSF3R/CXCL13/CXCR2/EDN3/S100A9 | 6 |
| BP | GO:0030595 | leukocyte chemotaxis | 236/18800 | 3.30883E-05 | 0.005121675 | 0.00435668 | CCL20/CSF3R/CXCL13/CXCR2/EDN3/FPR2/S100A9 | 7 |
| BP | GO:0002544 | chronic inflammatory response | 17/18800 | 3.52409E-05 | 0.005121675 | 0.00435668 | CXCL13/IDO1/S100A9 | 3 |
| BP | GO:0019730 | antimicrobial humoral response | 122/18800 | 0.000104776 | 0.013704723 | 0.011657728 | CXCL13/RPL30/S100A9/TF/WFDC13 | 5 |
| BP | GO:0045109 | intermediate filament organization | 68/18800 | 0.000133818 | 0.015912141 | 0.013535436 | KRT16/KRT20/KRT23/KRT80 | 4 |
| BP | GO:0034368 | protein-lipid complex remodeling | 30/18800 | 0.000203022 | 0.020427157 | 0.01737607 | APOC2/PLA2G2A/PLTP | 3 |
| BP | GO:0034369 | plasma lipoprotein particle remodeling | 30/18800 | 0.000203022 | 0.020427157 | 0.01737607 | APOC2/PLA2G2A/PLTP | 3 |
| BP | GO:0034367 | protein-containing complex remodeling | 32/18800 | 0.000246669 | 0.021822554 | 0.018563045 | APOC2/PLA2G2A/PLTP | 3 |
| BP | GO:0009913 | epidermal cell differentiation | 230/18800 | 0.000250259 | 0.021822554 | 0.018563045 | ACER1/KRT16/KRT80/OVOL1/SCEL/SFRP4 | 6 |
| BP | GO:0006898 | receptor-mediated endocytosis | 246/18800 | 0.000358644 | 0.027442351 | 0.023343445 | APOC2/C3/CXCR2/FPR2/SFRP4/TF | 6 |
| BP | GO:0045104 | intermediate filament cytoskeleton organization | 88/18800 | 0.000361714 | 0.027442351 | 0.023343445 | KRT16/KRT20/KRT23/KRT80 | 4 |
| BP | GO:0045103 | intermediate filament-based process | 89/18800 | 0.000377647 | 0.027442351 | 0.023343445 | KRT16/KRT20/KRT23/KRT80 | 4 |
| BP | GO:0008544 | epidermis development | 355/18800 | 0.000414644 | 0.028544991 | 0.02428139 | ACER1/EMP1/KRT16/KRT80/OVOL1/SCEL/SFRP4 | 7 |
| BP | GO:0072503 | cellular divalent inorganic cation homeostasis | 494/18800 | 0.000588441 | 0.038484067 | 0.032735924 | ATP2B2/CXCL13/CXCR2/EDN3/FPR2/MT1A/S100A9/TRPA1 | 8 |
| BP | GO:0050900 | leukocyte migration | 384/18800 | 0.000661133 | 0.039268345 | 0.033403058 | CCL20/CSF3R/CXCL13/CXCR2/EDN3/FPR2/S100A9 | 7 |
| BP | GO:0071827 | plasma lipoprotein particle organization | 45/18800 | 0.000680971 | 0.039268345 | 0.033403058 | APOC2/PLA2G2A/PLTP | 3 |
| BP | GO:0006575 | cellular modified amino acid metabolic process | 188/18800 | 0.000771445 | 0.039268345 | 0.033403058 | GGH/GSTA3/GSTA4/GSTA5/SULT1B1 | 5 |
| BP | GO:0006222 | UMP biosynthetic process | 11/18800 | 0.000777958 | 0.039268345 | 0.033403058 | CDA/CMPK1 | 2 |
| BP | GO:0009174 | pyrimidine ribonucleoside monophosphate biosynthetic process | 11/18800 | 0.000777958 | 0.039268345 | 0.033403058 | CDA/CMPK1 | 2 |
| BP | GO:0006805 | xenobiotic metabolic process | 108/18800 | 0.000784811 | 0.039268345 | 0.033403058 | GSTA3/GSTA4/GSTA5/SULT1B1 | 4 |
| BP | GO:0071825 | protein-lipid complex subunit organization | 48/18800 | 0.000823232 | 0.039268345 | 0.033403058 | APOC2/PLA2G2A/PLTP | 3 |
| BP | GO:0048259 | regulation of receptor-mediated endocytosis | 110/18800 | 0.000840607 | 0.039268345 | 0.033403058 | APOC2/C3/SFRP4/TF | 4 |
| BP | GO:0009410 | response to xenobiotic stimulus | 411/18800 | 0.000984036 | 0.043262718 | 0.036800815 | ABCC6/GSTA3/GSTA4/GSTA5/HMGCS2/SULT1B1/TRPA1 | 7 |
| BP | GO:0046916 | cellular transition metal ion homeostasis | 115/18800 | 0.000992264 | 0.043262718 | 0.036800815 | LCN2/MT1A/S100A9/TF | 4 |
| BP | GO:0055062 | phosphate ion homeostasis | 13/18800 | 0.001097829 | 0.043593598 | 0.037082273 | ABCC6/SFRP4 | 2 |
| BP | GO:0072506 | trivalent inorganic anion homeostasis | 13/18800 | 0.001097829 | 0.043593598 | 0.037082273 | ABCC6/SFRP4 | 2 |
| BP | GO:0048260 | positive regulation of receptor-mediated endocytosis | 53/18800 | 0.001099838 | 0.043593598 | 0.037082273 | C3/SFRP4/TF | 3 |
| BP | GO:0071715 | icosanoid transport | 54/18800 | 0.001161352 | 0.044677896 | 0.038004616 | ABCC6/PLA2G2A/PLA2G2D | 3 |
| BP | GO:0002864 | regulation of acute inflammatory response to antigenic stimulus | 14/18800 | 0.00127763 | 0.047746863 | 0.040615189 | C3/PLA2G2D | 2 |
| BP | GO:0006959 | humoral immune response | 317/18800 | 0.001346043 | 0.048906217 | 0.041601377 | C3/CXCL13/RPL30/S100A9/TF/WFDC13 | 6 |
| BP | GO:0006206 | pyrimidine nucleobase metabolic process | 15/18800 | 0.001470541 | 0.048925562 | 0.041617833 | CDA/CMPK1 | 2 |
| BP | GO:0009173 | pyrimidine ribonucleoside monophosphate metabolic process | 15/18800 | 0.001470541 | 0.048925562 | 0.041617833 | CDA/CMPK1 | 2 |
| BP | GO:0046049 | UMP metabolic process | 15/18800 | 0.001470541 | 0.048925562 | 0.041617833 | CDA/CMPK1 | 2 |
| BP | GO:0032103 | positive regulation of response to external stimulus | 442/18800 | 0.001496195 | 0.048925562 | 0.041617833 | C3/CXCL13/EDN3/FPR2/IDO1/PLA2G2A/S100A9 | 7 |
| BP | GO:0009130 | pyrimidine nucleoside monophosphate biosynthetic process | 16/18800 | 0.001676461 | 0.052209776 | 0.044411503 | CDA/CMPK1 | 2 |
| BP | GO:0051238 | sequestering of metal ion | 16/18800 | 0.001676461 | 0.052209776 | 0.044411503 | LCN2/S100A9 | 2 |
| BP | GO:0032496 | response to lipopolysaccharide | 333/18800 | 0.001728155 | 0.052568054 | 0.044716266 | ALPL/CXCL13/HMGCS2/IDO1/NR4A1/S100A9 | 6 |
| BP | GO:0006749 | glutathione metabolic process | 65/18800 | 0.00198455 | 0.057992672 | 0.049330641 | GSTA3/GSTA4/GSTA5 | 3 |
| BP | GO:0055076 | transition metal ion homeostasis | 139/18800 | 0.001995161 | 0.057992672 | 0.049330641 | LCN2/MT1A/S100A9/TF | 4 |
| BP | GO:0071677 | positive regulation of mononuclear cell migration | 66/18800 | 0.00207338 | 0.058490646 | 0.049754235 | CCL20/CXCL13/FPR2 | 3 |
| BP | GO:0002687 | positive regulation of leukocyte migration | 141/18800 | 0.002101728 | 0.058490646 | 0.049754235 | CCL20/CXCL13/EDN3/FPR2 | 4 |
| BP | GO:0045861 | negative regulation of proteolysis | 350/18800 | 0.002219293 | 0.060475727 | 0.051442816 | C3/CST4/NR4A1/SERPINB2/TM4SF20/WFDC13 | 6 |
| BP | GO:0050729 | positive regulation of inflammatory response | 145/18800 | 0.002326557 | 0.06144475 | 0.052267102 | C3/IDO1/PLA2G2A/S100A9 | 4 |
| BP | GO:0002237 | response to molecule of bacterial origin | 354/18800 | 0.002348805 | 0.06144475 | 0.052267102 | ALPL/CXCL13/HMGCS2/IDO1/NR4A1/S100A9 | 6 |
| BP | GO:0010951 | negative regulation of endopeptidase activity | 251/18800 | 0.002755418 | 0.069544011 | 0.059156623 | C3/CST4/NR4A1/SERPINB2/WFDC13 | 5 |
| BP | GO:0033555 | multicellular organismal response to stress | 73/18800 | 0.002764747 | 0.069544011 | 0.059156623 | HMGCS2/IDO1/TRPA1 | 3 |
| BP | GO:0006067 | ethanol metabolic process | 21/18800 | 0.002897728 | 0.071513731 | 0.060832137 | ADH7/SULT1B1 | 2 |
| BP | GO:0009129 | pyrimidine nucleoside monophosphate metabolic process | 22/18800 | 0.003179628 | 0.077017665 | 0.065513981 | CDA/CMPK1 | 2 |
| BP | GO:0010466 | negative regulation of peptidase activity | 262/18800 | 0.00331147 | 0.078341354 | 0.066639957 | C3/CST4/NR4A1/SERPINB2/WFDC13 | 5 |
| BP | GO:0061844 | antimicrobial humoral immune response mediated by antimicrobial peptide | 79/18800 | 0.003458174 | 0.078341354 | 0.066639957 | CXCL13/RPL30/S100A9 | 3 |
| BP | GO:0009220 | pyrimidine ribonucleotide biosynthetic process | 23/18800 | 0.003473852 | 0.078341354 | 0.066639957 | CDA/CMPK1 | 2 |
| BP | GO:0014821 | phasic smooth muscle contraction | 23/18800 | 0.003473852 | 0.078341354 | 0.066639957 | EDN3/SSTR2 | 2 |
| BP | GO:0006644 | phospholipid metabolic process | 388/18800 | 0.00369275 | 0.081866384 | 0.069638474 | APOC2/CWH43/FADS1/HMGCS2/PLA2G2A/PLA2G2D | 6 |
| BP | GO:0030216 | keratinocyte differentiation | 167/18800 | 0.003865204 | 0.083952759 | 0.071413219 | ACER1/KRT16/KRT80/SCEL | 4 |
| BP | GO:0071466 | cellular response to xenobiotic stimulus | 168/18800 | 0.003948176 | 0.083952759 | 0.071413219 | GSTA3/GSTA4/GSTA5/SULT1B1 | 4 |
| BP | GO:0050727 | regulation of inflammatory response | 394/18800 | 0.003979412 | 0.083952759 | 0.071413219 | C3/FPR2/IDO1/PLA2G2A/PLA2G2D/S100A9 | 6 |
| BP | GO:0002438 | acute inflammatory response to antigenic stimulus | 25/18800 | 0.004098885 | 0.084007051 | 0.071459402 | C3/PLA2G2D | 2 |
| BP | GO:0097006 | regulation of plasma lipoprotein particle levels | 84/18800 | 0.004110437 | 0.084007051 | 0.071459402 | APOC2/PLA2G2A/PLTP | 3 |
| BP | GO:0070098 | chemokine-mediated signaling pathway | 89/18800 | 0.004832764 | 0.097250088 | 0.082724403 | CCL20/CXCL13/CXCR2 | 3 |
| BP | GO:0031349 | positive regulation of defense response | 289/18800 | 0.005013576 | 0.099359969 | 0.084519143 | C3/FPR2/IDO1/PLA2G2A/S100A9 | 5 |
| MF | GO:0004364 | glutathione transferase activity | 26/18410 | 0.000139796 | 0.035088773 | 0.030460793 | GSTA3/GSTA4/GSTA5 | 3 |
| KEGG | hsa00983 | Drug metabolism - other enzymes | 80/8164 | 0.000108368 | 0.011487008 | 0.009353869 | CDA/CMPK1/GSTA3/GSTA4/GSTA5 | 5 |
| KEGG | hsa05150 | Staphylococcus aureus infection | 96/8164 | 0.000256425 | 0.013590539 | 0.011066774 | C3/FPR2/KRT16/KRT20/KRT23 | 5 |
| KEGG | hsa00982 | Drug metabolism - cytochrome P450 | 72/8164 | 0.000873247 | 0.030854737 | 0.025125009 | ADH7/GSTA3/GSTA4/GSTA5 | 4 |
| KEGG | hsa00980 | Metabolism of xenobiotics by cytochrome P450 | 78/8164 | 0.001179268 | 0.031250603 | 0.025447363 | ADH7/GSTA3/GSTA4/GSTA5 | 4 |
| KEGG | hsa04972 | Pancreatic secretion | 102/8164 | 0.003162227 | 0.06703921 | 0.054590022 | ATP2B2/CLCA2/PLA2G2A/PLA2G2D | 4 |
| KEGG | hsa00480 | Glutathione metabolism | 57/8164 | 0.004736037 | 0.082826362 | 0.067445499 | GSTA3/GSTA4/GSTA5 | 3 |
| KEGG | hsa04978 | Mineral absorption | 60/8164 | 0.005469665 | 0.082826362 | 0.067445499 | ATP2B2/MT1A/TF | 3 |
| KEGG | hsa05204 | Chemical carcinogenesis - DNA adducts | 69/8164 | 0.008064975 | 0.092423869 | 0.075260748 | GSTA3/GSTA4/GSTA5 | 3 |
| KEGG | hsa04915 | Estrogen signaling pathway | 138/8164 | 0.009195219 | 0.092423869 | 0.075260748 | KCNJ5/KRT16/KRT20/KRT23 | 4 |
| KEGG | hsa01524 | Platinum drug resistance | 73/8164 | 0.009415063 | 0.092423869 | 0.075260748 | GSTA3/GSTA4/GSTA5 | 3 |
| KEGG | hsa00592 | alpha-Linolenic acid metabolism | 25/8164 | 0.009695107 | 0.092423869 | 0.075260748 | PLA2G2A/PLA2G2D | 2 |
| KEGG | hsa00790 | Folate biosynthesis | 26/8164 | 0.01046308 | 0.092423869 | 0.075260748 | ALPL/GGH | 2 |
| KEGG | hsa00591 | Linoleic acid metabolism | 29/8164 | 0.012922326 | 0.09896438 | 0.080586685 | PLA2G2A/PLA2G2D | 2 |
| KEGG | hsa01240 | Biosynthesis of cofactors | 153/8164 | 0.013070767 | 0.09896438 | 0.080586685 | ALPL/CMPK1/GGH/IDO1 | 4 |
