# Supplementary material for: Identification of potential biomarkers and therapeutic targets for underactive bladder based on bioinformatics analysis and experimental validation
Source: PLoS One. 2025 Nov 6;20(11):e0335455. doi: 10.1371/journal.pone.0335455 (PMC12591491; doi:10.1371/journal.pone.0335455)
Supplement: S3 Table — (DOCX) [file pone.0335455.s003.docx]

| ***gene*** | ***Degree*** |
| --- | --- |
| ALDH1A1 | Degree: 26.0 |
| S100A9 | Degree: 22.0 |
| C3 | Degree: 22.0 |
| LCN2 | Degree: 21.0 |
| SERPINB2 | Degree: 17.0 |
| PLA2G2A | Degree: 17.0 |
| CXCR2 | Degree: 17.0 |
| IDO1 | Degree: 15.0 |
| CXCL13 | Degree: 15.0 |
| CCL20 | Degree: 14.0 |
| FPR2 | Degree: 14.0 |
| KRT20 | Degree: 14.0 |
| SCEL | Degree: 14.0 |
| HMGCS2 | Degree: 14.0 |
| KRT16 | Degree: 13.0 |
| ATP2B2 | Degree: 12.0 |
| CLEC4E | Degree: 11.0 |
| LGALS4 | Degree: 11.0 |
| CSF3R | Degree: 11.0 |
| TF | Degree: 10.0 |
| CLCA2 | Degree: 10.0 |
| KRT80 | Degree: 10.0 |
| ADH7 | Degree: 10.0 |
| CDA | Degree: 9.0 |
| SFRP4 | Degree: 9.0 |
| GSTA4 | Degree: 9.0 |
| NR4A1 | Degree: 8.0 |
| EMP1 | Degree: 8.0 |
| CWH43 | Degree: 8.0 |
| APOC2 | Degree: 8.0 |
| LILRB3 | Degree: 7.0 |
| PLTP | Degree: 7.0 |
| ANXA10 | Degree: 7.0 |
| TRPA1 | Degree: 7.0 |
| GSTA5 | Degree: 7.0 |
| SSTR2 | Degree: 6.0 |
| NXPH1 | Degree: 6.0 |
| SULT1B1 | Degree: 6.0 |
| GSTA3 | Degree: 6.0 |
| ABCC6 | Degree: 6.0 |
| KRT23 | Degree: 5.0 |
| KLK9 | Degree: 5.0 |
| F10 | Degree: 5.0 |
| CST4 | Degree: 5.0 |
| SCGB2A2 | Degree: 5.0 |
| OVOL1 | Degree: 5.0 |
| ACER1 | Degree: 5.0 |
| ALPL | Degree: 5.0 |
| GGH | Degree: 4.0 |
| PLA2G2D | Degree: 4.0 |
| PRR15 | Degree: 4.0 |
| MPP2 | Degree: 4.0 |
| NCAM2 | Degree: 4.0 |
| PRG4 | Degree: 4.0 |
| CRACD | Degree: 4.0 |
| SBSPON | Degree: 4.0 |
| MAB21L4 | Degree: 3.0 |
| SPDEF | Degree: 3.0 |
| WFDC13 | Degree: 3.0 |
| NQO2 | Degree: 3.0 |
| MT1A | Degree: 3.0 |
| FADS1 | Degree: 3.0 |
| CMPK1 | Degree: 3.0 |
| NAT8L | Degree: 3.0 |
| PSD2 | Degree: 3.0 |
| GPRC5A | Degree: 3.0 |
| EDN3 | Degree: 3.0 |
| CHRNB4 | Degree: 3.0 |
| PCDH10 | Degree: 2.0 |
| SCGB1D4 | Degree: 2.0 |
| RPL30 | Degree: 2.0 |
| PLEKHS1 | Degree: 2.0 |
| WDR66 | Degree: 2.0 |
| XKRX | Degree: 2.0 |
| NEB | Degree: 2.0 |
| KCNJ5 | Degree: 2.0 |
| SCGB1D2 | Degree: 1.0 |
| PDLIM2 | Degree: 1.0 |
| SPAG16 | Degree: 1.0 |
| FAM25A | Degree: 1.0 |
