# Supplementary material for: Identification of potential biomarkers and therapeutic targets for underactive bladder based on bioinformatics analysis and experimental validation
Source: PLoS One. 2025 Nov 6;20(11):e0335455. doi: 10.1371/journal.pone.0335455 (PMC12591491; doi:10.1371/journal.pone.0335455)
Supplement: S4 Table — (DOCX) [file pone.0335455.s004.docx]

| ***seed*** | ***TF*** |
| --- | --- |
| FPR2 | NKX3-1 |
| FPR2 | BRCA1 |
| FPR2 | FOXC1 |
| FPR2 | GATA2 |
| FPR2 | FOXL1 |
| FPR2 | MEF2A |
| FPR2 | HOXA5 |
| FPR2 | NFIC |
| FPR2 | NFYA |
| FPR2 | SRF |
| FPR2 | PPARG |
| FPR2 | GATA3 |
| FPR2 | RELA |
| FPR2 | TP53 |
| FPR2 | MAX |
| FPR2 | USF1 |
| FPR2 | USF2 |
| FPR2 | SREBF1 |
| FPR2 | PRDM1 |
| FPR2 | FOXF2 |
| FPR2 | TP63 |
| FPR2 | SREBF2 |
| FPR2 | NR3C1 |
| FPR2 | SOX5 |
| CLEC4E | PRRX2 |
| CLEC4E | JUND |
| CLEC4E | FOXC1 |
| CLEC4E | FOXL1 |
| CLEC4E | MEF2A |
| CLEC4E | HNF4A |
| CLEC4E | HINFP |
| CLEC4E | NFKB1 |
| CLEC4E | PAX2 |
| IDO1 | NKX3-2 |
| IDO1 | FOS |
| IDO1 | JUN |
| IDO1 | JUND |
| IDO1 | NFYA |
| IDO1 | HNF4A |
| IDO1 | HINFP |
| IDO1 | ELK1 |
| IDO1 | TP63 |
| IDO1 | RUNX2 |
| IDO1 | PAX2 |
| CSF3R | JUND |
| CSF3R | SRF |
| CSF3R | PPARG |
| CSF3R | IRF2 |
| CSF3R | PAX2 |
| C3 | FOXC1 |
| C3 | GATA2 |
| C3 | NFIC |
| C3 | PPARG |
| C3 | STAT1 |
| C3 | IRF2 |
| C3 | ESR1 |
| C3 | SREBF2 |
| CXCR2 | HOXA5 |
| CXCR2 | NFYA |
| CXCR2 | PPARG |
| CXCR2 | STAT3 |
| CXCR2 | ESR1 |
| CXCR2 | FOXA1 |
