# Supplementary material for: Identification of potential biomarkers and therapeutic targets for underactive bladder based on bioinformatics analysis and experimental validation
Source: PLoS One. 2025 Nov 6;20(11):e0335455. doi: 10.1371/journal.pone.0335455 (PMC12591491; doi:10.1371/journal.pone.0335455)
Supplement: S5 Table — (DOCX) [file pone.0335455.s005.docx]

| ***Id*** | ***Label*** | ***Degree*** | ***Betweenness*** |
| --- | --- | --- | --- |
| 718 | CXCR2 | 128 | 14020.21 |
| 3620 | CSF3R | 31 | 2878.51 |
| 2358 | C3 | 28 | 3212.82 |
| 26253 | CLEC4E | 19 | 1137.22 |
| 3579 | FPR2 | 12 | 798.16 |
| 1441 | IDO1 | 9 | 516.09 |
| 5468 | PPARG | 4 | 761.21 |
| MIMAT0000084 | hsa-miR-27a-3p | 4 | 430.3 |
| 2296 | FOXC1 | 3 | 588.26 |
| MIMAT0000460 | hsa-miR-194-5p | 3 | 302.5 |
| MIMAT0000081 | hsa-miR-25-3p | 3 | 256.16 |
| MIMAT0000255 | hsa-miR-34a-5p | 3 | 256.16 |
| MIMAT0003218 | hsa-miR-92b-3p | 3 | 256.16 |
| 4800 | NFYA | 3 | 245.47 |
| 3727 | JUND | 3 | 72.16 |
| 5076 | PAX2 | 3 | 72.16 |
| 2624 | GATA2 | 2 | 450.81 |
| 4782 | NFIC | 2 | 450.81 |
| 6721 | SREBF2 | 2 | 450.81 |
| 8626 | TP63 | 2 | 175.86 |
| MIMAT0000073 | hsa-miR-19a-3p | 2 | 160.6 |
| MIMAT0000074 | hsa-miR-19b-3p | 2 | 160.6 |
| MIMAT0000226 | hsa-miR-196a-5p | 2 | 160.6 |
| MIMAT0000425 | hsa-miR-130a-3p | 2 | 160.6 |
| MIMAT0000427 | hsa-miR-133a-3p | 2 | 160.6 |
| MIMAT0000684 | hsa-miR-302a-3p | 2 | 160.6 |
| MIMAT0004605 | hsa-miR-129-2-3p | 2 | 160.6 |
| 3660 | IRF2 | 2 | 108.05 |
| MIMAT0000069 | hsa-miR-16-5p | 2 | 108.05 |
| MIMAT0000101 | hsa-miR-103a-3p | 2 | 108.05 |
| 2099 | ESR1 | 2 | 103.05 |
| MIMAT0000082 | hsa-miR-26a-5p | 2 | 103.05 |
| MIMAT0001536 | hsa-miR-429 | 2 | 103.05 |
| MIMAT0000065 | hsa-let-7d-5p | 2 | 78.21 |
| MIMAT0000072 | hsa-miR-18a-5p | 2 | 78.21 |
| MIMAT0000092 | hsa-miR-92a-3p | 2 | 78.21 |
| MIMAT0000250 | hsa-miR-139-5p | 2 | 78.21 |
| MIMAT0001412 | hsa-miR-18b-5p | 2 | 78.21 |
| 2300 | FOXL1 | 2 | 59.25 |
| 4205 | MEF2A | 2 | 59.25 |
| 6722 | SRF | 2 | 45.88 |
| 3202 | HOXA5 | 2 | 30.77 |
| MIMAT0000445 | hsa-miR-126-3p | 2 | 30.77 |
| 3172 | HNF4A | 2 | 17.35 |
| 25988 | HINFP | 2 | 17.35 |
| 4824 | NKX3-1 | 1 | 0 |
| 51450 | PRRX2 | 1 | 0 |
| 579 | NKX3-2 | 1 | 0 |
| 672 | BRCA1 | 1 | 0 |
| 2353 | FOS | 1 | 0 |
| 3725 | JUN | 1 | 0 |
| 6774 | STAT3 | 1 | 0 |
| 2625 | GATA3 | 1 | 0 |
| 4790 | NFKB1 | 1 | 0 |
| 5970 | RELA | 1 | 0 |
| 7157 | TP53 | 1 | 0 |
| 4149 | MAX | 1 | 0 |
| 7391 | USF1 | 1 | 0 |
| 7392 | USF2 | 1 | 0 |
| 6720 | SREBF1 | 1 | 0 |
| 6772 | STAT1 | 1 | 0 |
| 2002 | ELK1 | 1 | 0 |
| 639 | PRDM1 | 1 | 0 |
| 2295 | FOXF2 | 1 | 0 |
| 3169 | FOXA1 | 1 | 0 |
| 860 | RUNX2 | 1 | 0 |
| 2908 | NR3C1 | 1 | 0 |
| 6660 | SOX5 | 1 | 0 |
| MIMAT0000062 | hsa-let-7a-5p | 1 | 0 |
| MIMAT0000063 | hsa-let-7b-5p | 1 | 0 |
| MIMAT0000064 | hsa-let-7c-5p | 1 | 0 |
| MIMAT0000066 | hsa-let-7e-5p | 1 | 0 |
| MIMAT0000067 | hsa-let-7f-5p | 1 | 0 |
| MIMAT0000068 | hsa-miR-15a-5p | 1 | 0 |
| MIMAT0000070 | hsa-miR-17-5p | 1 | 0 |
| MIMAT0000075 | hsa-miR-20a-5p | 1 | 0 |
| MIMAT0000076 | hsa-miR-21-5p | 1 | 0 |
| MIMAT0000080 | hsa-miR-24-3p | 1 | 0 |
| MIMAT0000083 | hsa-miR-26b-5p | 1 | 0 |
| MIMAT0000085 | hsa-miR-28-5p | 1 | 0 |
| MIMAT0000086 | hsa-miR-29a-3p | 1 | 0 |
| MIMAT0000087 | hsa-miR-30a-5p | 1 | 0 |
| MIMAT0000096 | hsa-miR-98-5p | 1 | 0 |
| MIMAT0000099 | hsa-miR-101-3p | 1 | 0 |
| MIMAT0000100 | hsa-miR-29b-3p | 1 | 0 |
| MIMAT0000103 | hsa-miR-106a-5p | 1 | 0 |
| MIMAT0000104 | hsa-miR-107 | 1 | 0 |
| MIMAT0000227 | hsa-miR-197-3p | 1 | 0 |
| MIMAT0000232 | hsa-miR-199a-3p | 1 | 0 |
| MIMAT0000252 | hsa-miR-7-5p | 1 | 0 |
| MIMAT0000254 | hsa-miR-10b-5p | 1 | 0 |
| MIMAT0000257 | hsa-miR-181b-5p | 1 | 0 |
| MIMAT0000259 | hsa-miR-182-5p | 1 | 0 |
| MIMAT0000261 | hsa-miR-183-5p | 1 | 0 |
| MIMAT0000264 | hsa-miR-203a-3p | 1 | 0 |
| MIMAT0000267 | hsa-miR-210-3p | 1 | 0 |
| MIMAT0000269 | hsa-miR-212-3p | 1 | 0 |
| MIMAT0000271 | hsa-miR-214-3p | 1 | 0 |
| MIMAT0000278 | hsa-miR-221-3p | 1 | 0 |
| MIMAT0000318 | hsa-miR-200b-3p | 1 | 0 |
| MIMAT0000414 | hsa-let-7g-5p | 1 | 0 |
| MIMAT0000415 | hsa-let-7i-5p | 1 | 0 |
| MIMAT0000416 | hsa-miR-1-3p | 1 | 0 |
| MIMAT0000417 | hsa-miR-15b-5p | 1 | 0 |
| MIMAT0000422 | hsa-miR-124-3p | 1 | 0 |
| MIMAT0000424 | hsa-miR-128-3p | 1 | 0 |
| MIMAT0000426 | hsa-miR-132-3p | 1 | 0 |
| MIMAT0000430 | hsa-miR-138-5p | 1 | 0 |
| MIMAT0000433 | hsa-miR-142-5p | 1 | 0 |
| MIMAT0000438 | hsa-miR-152-3p | 1 | 0 |
| MIMAT0000440 | hsa-miR-191-5p | 1 | 0 |
| MIMAT0000449 | hsa-miR-146a-5p | 1 | 0 |
| MIMAT0000450 | hsa-miR-149-5p | 1 | 0 |
| MIMAT0000461 | hsa-miR-195-5p | 1 | 0 |
| MIMAT0000617 | hsa-miR-200c-3p | 1 | 0 |
| MIMAT0000646 | hsa-miR-155-5p | 1 | 0 |
| MIMAT0000680 | hsa-miR-106b-5p | 1 | 0 |
| MIMAT0000681 | hsa-miR-29c-3p | 1 | 0 |
| MIMAT0000685 | hsa-miR-34b-5p | 1 | 0 |
| MIMAT0000686 | hsa-miR-34c-5p | 1 | 0 |
| MIMAT0000688 | hsa-miR-301a-3p | 1 | 0 |
| MIMAT0000691 | hsa-miR-130b-3p | 1 | 0 |
| MIMAT0000724 | hsa-miR-372-3p | 1 | 0 |
| MIMAT0000726 | hsa-miR-373-3p | 1 | 0 |
| MIMAT0000728 | hsa-miR-375 | 1 | 0 |
| MIMAT0000732 | hsa-miR-378a-3p | 1 | 0 |
| MIMAT0000734 | hsa-miR-380-5p | 1 | 0 |
| MIMAT0000737 | hsa-miR-382-5p | 1 | 0 |
| MIMAT0000759 | hsa-miR-148b-3p | 1 | 0 |
| MIMAT0000761 | hsa-miR-324-5p | 1 | 0 |
| MIMAT0000765 | hsa-miR-335-5p | 1 | 0 |
| MIMAT0000773 | hsa-miR-346 | 1 | 0 |
| MIMAT0001341 | hsa-miR-424-5p | 1 | 0 |
| MIMAT0001413 | hsa-miR-20b-5p | 1 | 0 |
| MIMAT0001541 | hsa-miR-449a | 1 | 0 |
| MIMAT0001635 | hsa-miR-452-5p | 1 | 0 |
| MIMAT0002174 | hsa-miR-484 | 1 | 0 |
| MIMAT0002830 | hsa-miR-520f-3p | 1 | 0 |
| MIMAT0002859 | hsa-miR-516b-5p | 1 | 0 |
| MIMAT0002872 | hsa-miR-501-5p | 1 | 0 |
| MIMAT0002888 | hsa-miR-532-5p | 1 | 0 |
| MIMAT0003249 | hsa-miR-584-5p | 1 | 0 |
| MIMAT0003294 | hsa-miR-625-5p | 1 | 0 |
| MIMAT0003297 | hsa-miR-628-3p | 1 | 0 |
| MIMAT0003327 | hsa-miR-449b-5p | 1 | 0 |
| MIMAT0003880 | hsa-miR-671-5p | 1 | 0 |
| MIMAT0003885 | hsa-miR-454-3p | 1 | 0 |
| MIMAT0003886 | hsa-miR-769-5p | 1 | 0 |
| MIMAT0003887 | hsa-miR-769-3p | 1 | 0 |
| MIMAT0003888 | hsa-miR-766-3p | 1 | 0 |
| MIMAT0004509 | hsa-miR-93-3p | 1 | 0 |
| MIMAT0004563 | hsa-miR-199b-3p | 1 | 0 |
| MIMAT0004589 | hsa-miR-30b-3p | 1 | 0 |
| MIMAT0004601 | hsa-miR-145-3p | 1 | 0 |
| MIMAT0004697 | hsa-miR-151a-5p | 1 | 0 |
| MIMAT0004699 | hsa-miR-148b-5p | 1 | 0 |
| MIMAT0004748 | hsa-miR-423-5p | 1 | 0 |
| MIMAT0004762 | hsa-miR-486-3p | 1 | 0 |
| MIMAT0004780 | hsa-miR-532-3p | 1 | 0 |
| MIMAT0004925 | hsa-miR-876-3p | 1 | 0 |
| MIMAT0004945 | hsa-miR-744-5p | 1 | 0 |
| MIMAT0004948 | hsa-miR-885-3p | 1 | 0 |
| MIMAT0004949 | hsa-miR-877-5p | 1 | 0 |
| MIMAT0004984 | hsa-miR-941 | 1 | 0 |
| MIMAT0005572 | hsa-miR-1225-5p | 1 | 0 |
| MIMAT0005792 | hsa-miR-320b | 1 | 0 |
| MIMAT0005793 | hsa-miR-320c | 1 | 0 |
| MIMAT0005797 | hsa-miR-1301-3p | 1 | 0 |
| MIMAT0013517 | hsa-miR-2682-5p | 1 | 0 |
| MIMAT0015378 | hsa-miR-3065-3p | 1 | 0 |
| MIMAT0018444 | hsa-miR-642b-3p | 1 | 0 |
| MIMAT0019064 | hsa-miR-4525 | 1 | 0 |
| MIMAT0019232 | hsa-miR-4423-5p | 1 | 0 |
| MIMAT0019737 | hsa-miR-4664-5p | 1 | 0 |
| MIMAT0019776 | hsa-miR-1343-3p | 1 | 0 |
| MIMAT0021043 | hsa-miR-5010-5p | 1 | 0 |
| MIMAT0022714 | hsa-miR-766-5p | 1 | 0 |
| MIMAT0026559 | hsa-miR-487a-5p | 1 | 0 |
| MIMAT0026637 | hsa-miR-1296-3p | 1 | 0 |
| MIMAT0026719 | hsa-miR-889-5p | 1 | 0 |
| MIMAT0031893 | hsa-miR-181b-2-3p | 1 | 0 |
| MIMAT0000510 | hsa-miR-320a-3p | 1 | 0 |
