# Supplementary material for: Identification of potential biomarkers and therapeutic targets for underactive bladder based on bioinformatics analysis and experimental validation
Source: PLoS One. 2025 Nov 6;20(11):e0335455. doi: 10.1371/journal.pone.0335455 (PMC12591491; doi:10.1371/journal.pone.0335455)
Supplement: S6 Table — (DOCX) [file pone.0335455.s006.docx]

| ***gene*** | ***drug*** | ***regulatory approval*** | ***indication*** | ***interaction score*** |
| --- | --- | --- | --- | --- |
| CXCR2 | BEVACIZUMAB | Approved | antineoplastic agent | 0.1450105515524598 |
| CXCR2 | DANIRIXIN | Not Approved |  | 2.900211031049196 |
| CXCR2 | AZD-5122 | Not Approved |  | 2.900211031049196 |
| CXCR2 | CLOTRIMAZOLE | Approved |  | 0.07838408192024855 |
| CXCR2 | ALPHA-TOCOPHEROL | Not Approved |  | 0.5273110965543993 |
| CXCR2 | NAVARIXIN | Not Approved | for treatment of chronic obstructive pulmonary disorder (COPD) | 1.160084412419678 |
| CXCR2 | LADARIXIN | Not Approved |  | 1.450105515524598 |
| CXCR2 | GENISTEIN | Approved |  | 0.1000072769327309 |
| CXCR2 | ACETYLCYSTEINE | Approved |  | 0.3866948041398929 |
| CXCR2 | ELUBRIXIN | Not Approved |  | 2.900211031049196 |
| CXCR2 | VIMNERIXIN | Not Approved |  | 2.900211031049196 |
| CXCR2 | REPARIXIN | Not Approved |  | 1.933474020699464 |
| CXCR2 | CANOCTAKIN | Not Approved | immunosuppressant,antiinflammatory agent | 1.450105515524598 |
| CXCR2 | MECHLORETHAMINE | Approved |  | 0.5273110965543993 |
| CXCR2 | IBUPROFEN, SODIUM SALT | Approved | NSAID | 0.07436438541151785 |
| CXCR2 | SX-682 | Not Approved |  | 1.450105515524598 |
| CXCR2 | CYCLOPHOSPHAMIDE ANHYDROUS | Approved |  | 0.07161014891479497 |
| CXCR2 | NAVARIXIN | Not Approved |  | 4.350316546573794 |
| FPR2 | ALVELESTAT | Not Approved |  | 2.175158273286897 |
| FPR2 | CHEMBL:CHEMBL1290365 | Not Approved |  | 4.350316546573794 |
| FPR2 | AG-26 | Not Approved |  | 4.350316546573794 |
| FPR2 | FMET-LEU-PHE | Not Approved |  | 2.900211031049196 |
| FPR2 | ALPHA 1-ANTITRYPSIN | Approved | Enzyme Replacement Agents | 1.450105515524598 |
| FPR2 | CHEMBL:CHEMBL1290139 | Not Approved |  | 8.700633093147589 |
| FPR2 | COMPOUND 10L [PMID: 24556381] | Not Approved |  | 4.350316546573794 |
| FPR2 | COMPOUND 10F [PMID: 24556381] | Not Approved |  | 4.350316546573794 |
| FPR2 | COMPOUND 4G [PMID: 22595175] | Not Approved |  | 1.450105515524598 |
| FPR2 | POSTSTATIN | Not Approved |  | 1.450105515524598 |
| FPR2 | BAY-678 | Not Approved |  | 4.350316546573794 |
| FPR2 | SIVELESTAT | Not Approved | for treatment of acute lung injury associated with systemic inflammatory response syndrome (SIRS) | 1.450105515524598 |
| CSF3R | TBO-FILGRASTIM | Approved | for treatment of neutropenia | 3.866948041398927 |
| CSF3R | TRAMETINIB DIMETHYL SULFOXIDE | Approved | antineoplastic agent | 0.1234132353637955 |
| CSF3R | PLX7486 | Not Approved |  | 0.6444913402331547 |
| CSF3R | PEGFILGRASTIM | Approved | Antineutropenic Agents,for treatment of neutropenia | 3.866948041398927 |
| CSF3R | AZD1480 | Not Approved |  | 0.4833685051748659 |
| CSF3R | PLX5622 | Not Approved |  | 1.288982680466309 |
| CSF3R | 2-TERT-BUTYL-9-FLUORO-1,6-DIHYDROBENZO[H]IMIDAZO[4,5-F]ISOQUINOLIN-7-ONE | Not Approved |  | 0.7733896082797855 |
| CSF3R | LENOGRASTIM | Approved |  | 0.1757703655181331 |
| CSF3R | RUXOLITINIB | Approved | antiinflammatory agent,antineoplastic agent | 1.812631894405747 |
| CSF3R | TOFACITINIB | Approved |  | 0.2762105743856377 |
| CSF3R | BENEGRASTIM | Approved |  | 1.933474020699463 |
| CSF3R | COMPOUND 1 [PMID: 16821802] | Not Approved |  | 1.933474020699463 |
| CSF3R | CSL-324 | Not Approved |  | 1.933474020699463 |
| CSF3R | MOMELOTINIB | Approved |  | 0.2974575416460714 |
| CSF3R | GLYCOPEG-GCSF | Not Approved | for treatment of neutropenia | 1.933474020699463 |
| CSF3R | BALUGRASTIM | Not Approved |  | 1.933474020699463 |
| CSF3R | IMATINIB | Approved | antineoplastic agent | 0.05370761168609622 |
| CSF3R | IBRUTINIB | Approved | antineoplastic agent | 0.1137337659234978 |
| CSF3R | LIPEGFILGRASTIM | Approved |  | 1.933474020699463 |
| CSF3R | REGRAMOSTIM | Not Approved |  | 1.933474020699463 |
| CSF3R | DASATINIB ANHYDROUS | Approved | antineoplastic agent | 0.1017617905631296 |
| CSF3R | BLZ-945 | Not Approved |  | 0.9667370103497319 |
| CSF3R | PEXIDARTINIB | Approved |  | 0.1381052871928188 |
| CSF3R | EFLAPEGRASTIM | Approved |  | 0.9667370103497319 |
| CSF3R | ANTI-C-FMS MONOCLONAL ANTIBODY AMG 820 | Not Approved |  | 0.9667370103497319 |
| CSF3R | PEGTEOGRASTIM | Not Approved |  | 1.933474020699463 |
| CSF3R | MAXY-G34 | Not Approved | for treatment of chemotherapy-induced neutropenia | 3.866948041398927 |
| IDO1 | CHEMBL:CHEMBL1224312 | Not Approved |  | 7.457685508412219 |
| IDO1 | LINRODOSTAT | Not Approved |  | 7.457685508412219 |
| IDO1 | 2-MERCAPTOBENZOTHIAZOLE | Not Approved |  | 0.9322106885515273 |
| IDO1 | PHENYLHYDRAZINE | Not Approved |  | 3.728842754206109 |
| IDO1 | PEGINTERFERON ALFA-2A | Approved | Antineoplastic Agents; Immunomodulatory Agents,for treatment of hepatitis B and C | 0.2571615692555937 |
| IDO1 | TRYPTOPHAN | Approved |  | 0.4661053442757636 |
| IDO1 | IDO-1 INHIBITOR LY3381916 | Not Approved |  | 3.728842754206109 |
| IDO1 | CHEMBL:CHEMBL1668301 | Not Approved |  | 7.457685508412219 |
| IDO1 | KHK2455 | Not Approved |  | 3.728842754206109 |
| IDO1 | CHEMBL:CHEMBL14145 | Not Approved |  | 7.457685508412219 |
| IDO1 | EPACADOSTAT | Not Approved |  | 3.728842754206109 |
| IDO1 | INDOXIMOD PRODRUG NLG802 | Not Approved |  | 3.728842754206109 |
| IDO1 | PF-06840003 | Not Approved |  | 1.864421377103054 |
| IDO1 | CHEMBL:CHEMBL1933308 | Not Approved |  | 7.457685508412219 |
| C3 | CA2+ | Not Approved |  | 0.1450105515524598 |
| C3 | OCTANOL | Not Approved |  | 0.2762105743856377 |
| C3 | AL-78898A | Not Approved | for treatment of age-related macular degeneration | 5.800422062098392 |
| C3 | AMY-101 | Not Approved |  | 5.800422062098392 |
| C3 | PEGCETACOPLAN | Approved |  | 2.900211031049196 |
| C3 | CARBENOXOLONE | Not Approved |  | 0.241684252587433 |
| C3 | FLUFENAMIC ACID | Not Approved |  | 0.1526426858446945 |
| C3 | CLOZAPINE | Approved | Antipsychotic Agents | 0.1195963311772864 |
| C3 | COMPSTATIN | Not Approved |  | 11.60084412419678 |
