# Supplementary figures and images for: Identification of potential biomarkers and therapeutic targets for underactive bladder based on bioinformatics analysis and experimental validation
Source: PLoS One. 2025 Nov 6;20(11):e0335455. doi: 10.1371/journal.pone.0335455 (PMC12591491; doi:10.1371/journal.pone.0335455)

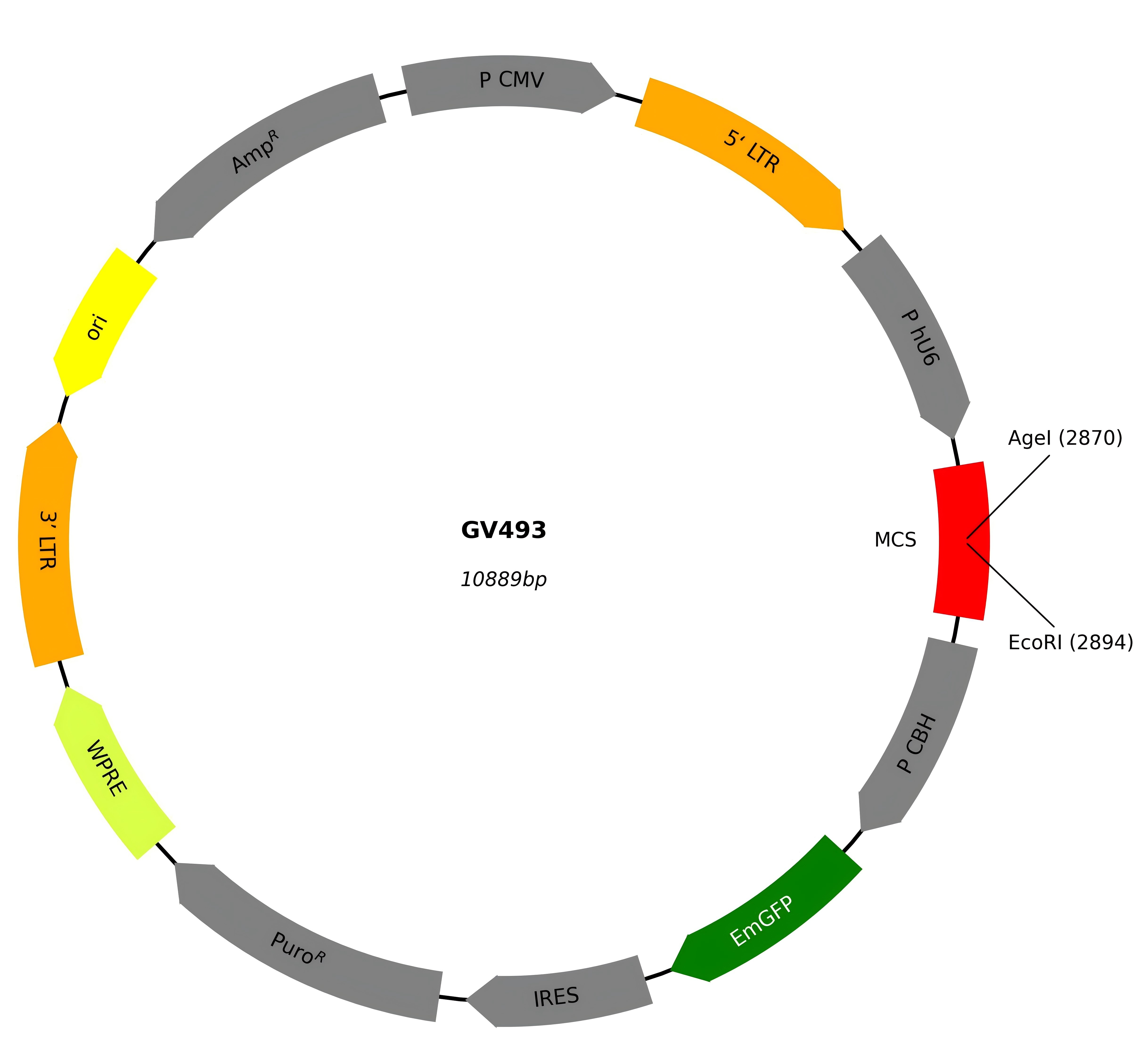

Supplement: S1 Fig — (JPG) [file pone.0335455.s007.jpg]

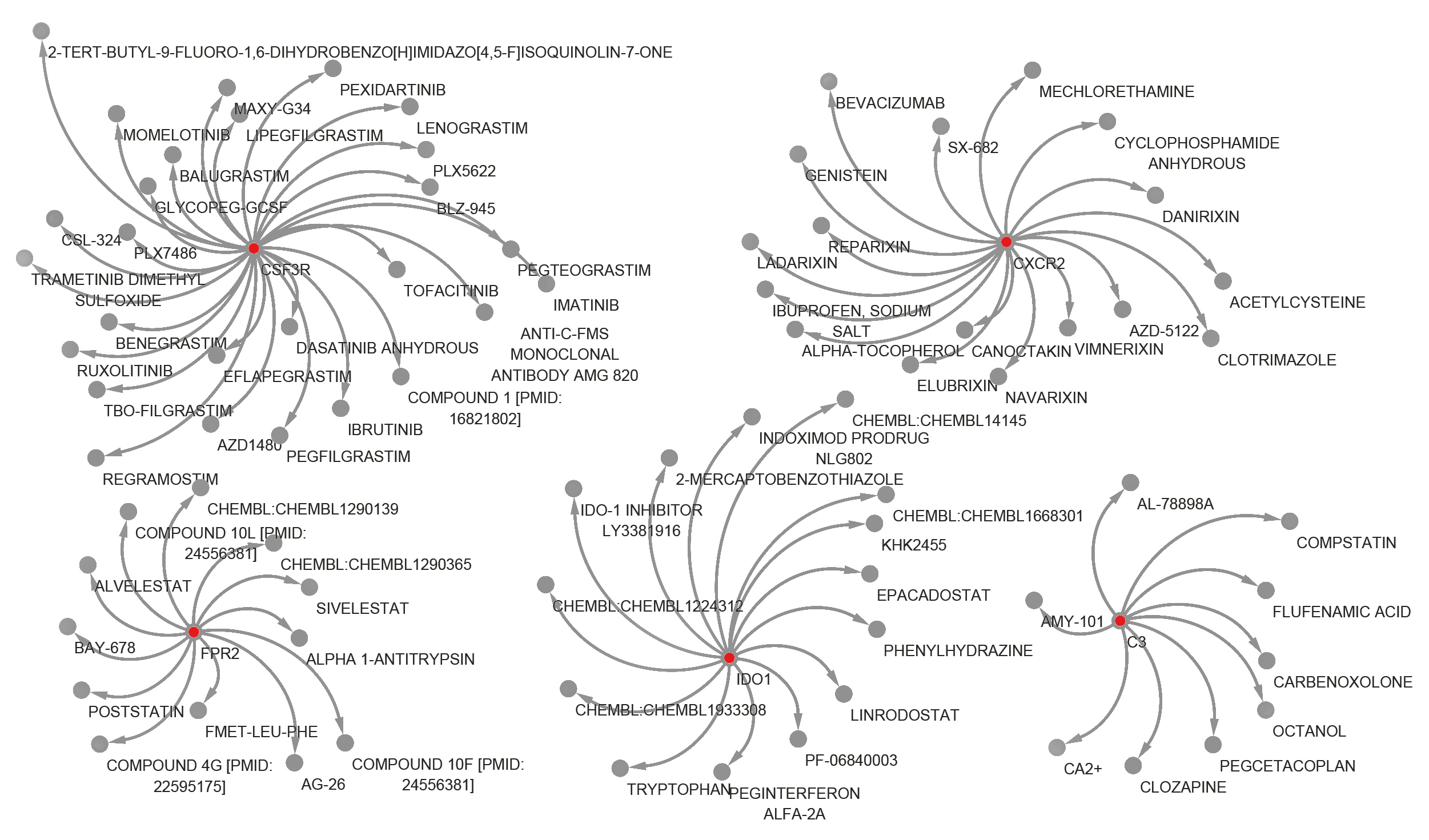

Supplement: S2 Fig — Red nodes represent hub genes, gray nodes represent corresponding drugs or molecular compounds. (JPG) [file pone.0335455.s008.jpg]
